# LDHA induces EMT gene transcription and regulates autophagy to promote the metastasis and tumorigenesis of papillary thyroid carcinoma

**DOI:** 10.1038/s41419-021-03641-8

**Published:** 2021-04-01

**Authors:** Xiukun Hou, Xianle Shi, Wei Zhang, Dapeng Li, Linfei Hu, Jihong Yang, Jingzhu Zhao, Songfeng Wei, Xi Wei, Xianhui Ruan, Xiangqian Zheng, Ming Gao

**Affiliations:** 1 Department of Thyroid and Neck Cancer, Tianjin Medical University Cancer Institute and Hospital, National Clinical Research Center for Cancer, Key Laboratory of Cancer Prevention and Therapy, Tianjin’s Clinical Research Center for Cancer, Tianjin, 300060 China; 2 Department of Medicine, Columbia Center for Human Development, Columbia University Irving Medical Center, New York, NY 10032 USA; 3 Department of Diagnostic and Therapeutic Ultrasonography, Tianjin Medical University Cancer Institute and Hospital, National Clinical Research Center for Cancer, Key Laboratory of Cancer Prevention and Therapy, Tianjin’s Clinical Research Center for Cancer, Tianjin, 300060 China

**Keywords:** Thyroid cancer, Target validation

## Abstract

Papillary thyroid carcinoma (PTC) is one of the most common kinds of endocrine-related cancer and has a heterogeneous prognosis. Metabolic reprogramming is one of the hallmarks of cancers. Aberrant glucose metabolism is associated with malignant biological behavior. However, the functions and mechanisms of glucose metabolism genes in PTC are not fully understood. Thus, data from The Cancer Genome Atlas database were analyzed, and lactate dehydrogenase A (LDHA) was determined to be a potential novel diagnostic and therapeutic target for PTCs. The research objective was to investigate the expression of LDHA in PTCs and to explore the main functions and relative mechanisms of LDHA in PTCs. Higher expression levels of LDHA were found in PTC tissues than in normal thyroid tissues at both the mRNA and protein levels. Higher expression levels of LDHA were correlated with aggressive clinicopathological features and poor prognosis. Moreover, we found that LDHA not only promoted PTC migration and invasion but also enhanced tumor growth both in vitro and in vivo. In addition, we revealed that the metabolic products of LDHA catalyzed induced the epithelial–mesenchymal transition process by increasing the relative gene H3K27 acetylation. Moreover, LDHA knockdown activated the AMPK pathway and induced protective autophagy. An autophagy inhibitor significantly enhanced the antitumor effect of FX11. These results suggested that LDHA enhanced the cell metastasis and proliferation of PTCs and may therefore become a potential therapeutic target for PTCs.

## Introduction

Papillary thyroid carcinoma (PTC) is the most common endocrine malignancy^1–3^. Although the 10-year overall survival (OS) rate of PTC is >95%, in some cases, PTC show dedifferentiation, which increases invasiveness and results in a poor prognosis^4^. However, the molecular mechanism of the pathology of PTC is still unclear and there are only a few treatment options available for PTC^5^. Therefore, biomarkers and potential therapeutic targets are needed to improve the diagnosis and treatment of PTC patients.

Metabolic reprogramming is common in tumors and has been accepted as one of the main hallmarks of all cancers^6,7^. Even when oxygen is sufficient, tumor cells prefer to use aerobic glycolysis to produce ATP, which is known as the Warburg effect^8,9^. Lactate dehydrogenase A (LDHA) is an enzyme that catalyzes the final step of the Warburg effect by converting the pyruvate and NADH to lactate. This leads to an acidic microenvironment for the tumor, contributing to epithelial–mesenchymal transition (EMT) and metastasis^10,11^. Upregulated LDHA has been reported in many types of tumors and is significantly correlated with poor prognosis^12,13^. The reduction in LDHA significantly inhibited cellular transformation and markedly delayed tumor formation^14–20^. In a breast cancer model, LDHA knockdown attenuated glycolysis and decreased tumor growth significantly by impacting mitochondrial physiology^21^. The suppression of LDHA attenuated the progression of glioblastoma through the downregulation of the Warburg effect^16^. In mouse models of lung cancer, targeting LDHA significantly inhibited tumorigenesis and tumor progression^20^. However, the molecular mechanisms of how LDHA contributes to tumor progression and tumor metastasis in PTC have not yet been investigated.

Dysregulation of histone modification, such as methylation, acetylation, and phosphorylation levels, occurs in various cancers, including PTC^22,23^. Histone acetylation is a reversible dynamic process that is achieved by histone acetyltransferases (HATs) and histone deacetylases. In recent years, an increasing number of studies have focused on histone acetylation for its role in changing the nucleosomal conformation and modulating chromatin structure and gene expression in cancers^24^. HATs transfer acetyl-coenzyme A (acetyl-CoA) to the terminus of histone amino acids and relax the structure of chromatin, which increases the accessibility of DNA and promotes gene transcription. As a key glucose metabolite, acetyl-CoA fuels the mitochondrial TCA cycle for ATP production through oxidative phosphorylation. Low intracellular acetyl-CoA decreases histone acetylation, which links glucose metabolism with chromatin remodeling^25,26^. Studies have shown that the absence of LDHA, an enzyme involved in glucose metabolism, decreases intracellular acetyl-CoA levels and diminishes histone acetylation^27^. However, whether LDHA affects histone acetylation levels in thyroid carcinoma is still unknown.

As one of the most important sensors of cellular energy, AMP-activated protein kinase (AMPK) coordinates various cellular pathways to maintain the balance of energy and nutrition metabolism^28,29^. As a metabolic sensor, AMPK allows for adaptive changes in metabolic coordination, cell growth, apoptosis, and autophagy^30^. It has been reported that the activation of AMPK, directly and indirectly, inhibits tumor growth by blocking the MAPK and PI3K-AKT signaling pathways^28,29^. The activation of AMPK reverses the Warburg effect from aerobic glycolysis to oxidative phosphorylation, but the relationship between LDHA and AMPK signaling has not been clarified.

Autophagy is a process of the cell that removes dysfunctional or unnecessary components^31^. Studies have shown that autophagy is a tightly regulated process and defects in autophagy have been closely associated with many human cancers^32,33^. It can be induced under stressful conditions, such as low cellular energy charge or the deprivation of essential nutrients^34^. As an enzyme involved in energy metabolism, LDHA was reported to induce prosurvival autophagy in tamoxifen-resistant breast cancer^35^. However, the functions of LDHA and autophagy in PTC are still largely unknown.

Although previous studies have shown that targeting LDHA attenuates tumor progression and metastasis, the molecular mechanism is still largely unknown. Here, we showed that the reduction in LDHA leads to the deregulation of intracellular acetyl-CoA, which reduces the histone acetylation of EMT-related genes in PTCs. In addition, the reduction in LDHA inhibited cell proliferation and promoted cell apoptosis via the AMPK pathway. Furthermore, we applied the combination of FX11 (a drug-like small molecule inhibitor of LDHA) and the autophagy inhibitor hydroxychloroquine (HCQ) in PTCs and found that HCQ could significantly enhance the effect of FX11 on PTCs in vitro and in vivo.

## Materials and methods

### Clinical data and tissue samples

Tissue microarrays including samples from 185 patients with complete medical record information were used in this research. All of the patients were diagnosed with PTC at Tianjin Medical University Cancer Institute and Hospital from January 2013 to June 2013. Twenty-eight matched fresh tissues of PTCs and adjacent normal thyroid follicular tissues were collected. Total RNA was extracted from 20 matched tissue samples, and total protein was extracted from eight matched tissue samples. The research was performed with the approval of the Ethics Committee of Tianjin Medical University Cancer Institute and Hospital. Informed consent was obtained for experimentation with human subjects.

### Immunohistochemistry (IHC)

The tissue microarrays of the PTC patients were subjected to immunohistochemical staining for LDHA according to a standard protocol. The signal was visualized with the DAB Substrate Kit (MaiXin Bio, China). The staining scores were evaluated by two experienced pathologists as follows: staining score = staining intensity + percentage of positive tumor cells. The staining intensity was regarded as 0 (no staining); 1 (weak, light yellow); 2 (moderate, light brown); or 3 (strong, brown). The percentage of positive cells was regarded as 0 (<5%), 1 (5–25%), 2 (25–50%), 3 (51–75%), or 4 (>75%). The specimens were divided into four levels: scores of 0–1 (−), a score of 2 (+), scores of 3–4 (++), and scores of more than 5 (+++). High expression levels of LDHA were defined as scores of 3–4 (++) and >5 (+++). For Ki-67 staining, positively stained cells were counted in five files and the total number of positive cells was calculated.

### Total RNA extraction and quantitative real-time PCR

Total RNA from thyroid cancer cells and fresh tissues was isolated by TRIzol reagent (Invitrogen, Carlsbad, USA), and cDNA was reverse transcribed with Prime Script RT Master Mix (Takara, Kyoto, Japan). Quantitative real-time PCR was performed with SYBR Premix Ex Taq II (Takara, Kyoto, Japan) and specific primers. The sequences of the primers are shown in Supplementary Table 3.

### Western blotting

Proteins from cells and tissues were extracted with radioimmunoprecipitation assay buffer (Solarbio, Beijing, China) according to the protocol. The concentration of protein samples was quantified by the BCA assay. Samples were run on 8–12% sodium dodecyl sulfate polyacrylamide gel electrophoresis gels and transferred to polyvinylidene difluoride membranes. Then, the cells were incubated with primary antibodies and peroxidase-conjugated anti-mouse or anti-rabbit IgG antibodies. Finally, the cells were visualized with ECL plus reagents (Cell Signaling Technology, Danvers, MA, USA).

### Cell culture

One normal thyroid follicular epithelial cell line (Nthy-ori 3–1), four PTC cell lines (K-1, KTC-1, TPC-1, and B-CPAP), one anaplastic thyroid carcinoma cell line (CAL-62), and one medullary thyroid carcinoma cell line (TT) were used in this study. Nthy-ori 3–1, K-1, and TPC-1 cells were purchased from the American Type Culture Collection (USA), and the other cell lines were purchased from the Chinese Academy of Sciences (Shanghai, China). All the cell lines were cultured in Roswell Park Memorial Institute Medium-1640 medium or Dulbecco’s Modified Eagle Medium (Gibco, USA) supplemented with 10% fetal bovine serum (FBS) and 1% penicillin and streptomycin and maintained in a humidified atmosphere with 5% CO_2_ at 37 °C.

### Transfection and lentiviral infection

For TGFβR1 and AMPK knockdown, three synthesized duplex RNAi oligos purchased from Sigma-Aldrich were transfected into cells using RNAiMAX (Invitrogen). RFP-LC3 plasmid was got from GeneChem (Shanghai, China) and transfected with Lipofectamine 3000 reagent (Invitrogen, USA).

Lentivirus vectors for LDHA knockdown and overexpression (shLDHA and OE-LDHA) were purchased from GeneChem (Shanghai, China). Polybrene (Sigma-Aldrich, Germany) was used to increase the efficiency of infection. Puromycin (2 μg/ml) was added to select the infected cells.

### Reagents, drugs, and antibodies

The following primary antibodies were used in the research: anti-FLAG, anti-LDHA, anti-Smad3, anti-phospho-Smad3 (anti-p-Smad3), anti-E-cadherin, anti-N-cadherin, anti-Slug, anti-AMPK, anti-phospho-AMPK (anti-p-AMPK), anti-mTOR, anti-phospho-mTOR (anti-p-mTOR), anti-ULK1, anti-phospho-ULK1 (anti-p-ULK1(Ser 555)), anti-LC3B, anti-p62, anti-cleaved caspase 3, anti-Ki-67, anti-Tri-Methyl-Histone H3 (Lys27), and anti-Tri-Methyl-Histone H3 (Lys36) (Cell Signaling Technology, USA). Anti-β-actin (GeneTex, USA). The LDHA inhibitor FX11 was purchased from Sigma-Aldrich (Darmstadt, Germany). HCQ was purchased from Selleck Chemicals (Houston, USA).

### Transwell of migration and invasion assay

Cell migration and invasion assays were performed with polycarbonate membrane filter inserts with 8-μM pores (Merck Millipore, USA) in 24-well chamber plates. The transwell chambers were precoated with (invasion assay) or without (migration assay) Matrigel (BD Biosciences, USA). In brief, the bottom chamber was filled with 500 μL of culture medium containing 10% FBS, and the upper chamber was filled with 200 μL of 2 × 10^4^ cell culture medium without FBS. The cells were incubated for 24 h with 5% CO_2_ at 37 °C. Then, cells at the bottom surface of the membrane were fixed in 4% polyoxymethylene for 30 min and stained with 0.1% crystal violet for 20 min. The mean numbers of five preselected microscopic fields were counted.

### Wound-healing assay

After LDHA knockdown and overexpression, 5 × 10^6^ cells were seeded in six-well plates. Then, the plates were scratched with 10 μL pipette tips and incubated at 37°C and 5% CO_2_ for 24 h. The wounded areas were observed and photographed under an inverted microscope.

### RNA-seq analysis

Total RNA was extracted with TRIzol reagent (Invitrogen, USA). cDNA libraries were generated and sequenced on an Illumina NovaSeq 6000 platform. RNA-Seq reads were trimmed using Trim Galore (v0.5.0). The trimmed data were aligned to the human hg19 genome using STAR (v2.7.0 f), and the aligned bam files were sorted by name using the parameter -n. We used HTSeq software (v0.11.2) and the hg19 annotation file from GENCODE (v19) to count the reads for each gene using the parameters -r name -f bam and BioMart to retrieve the corresponding gene names. Finally, the read counts were normalized with the trimmed mean of M-values (TMM) method for differential expression analysis using edgeR (v3.26.8). Sequencing data were deposited in the Gene Expression Omnibus (GSE167971).

### Gene Ontology (GO) analysis and gene set enrichment analysis (GSEA)

The Database for Annotation, Visualization and Integrated Discovery (DAVID) web tool was used to perform GO analysis. Significant enrichment was defined as *p* < 0.05. GSEA was performed with the GSEA stand-alone desktop program. Significantly enriched molecular function terms were defined by *p* < 0.05.

### Acetyl-CoA assay

For the measurement of cytosolic acetyl-CoA, cells were lysed on ice for 10 minutes. The lysates were spun down at 20,000 × *g* for 10 minutes at 4°C. Then the supernatants were measured with an acetyl-CoA assay kit (Sigma-Aldrich, Germany).

### Chromatin immunoprecipitation (ChIP)-Seq data analysis

ChIP-Seq data were downloaded from the Gene Expression Omnibus (GSE120177). ChIP-Seq peaks were generated by the peak-finding algorithm model-based analysis for ChIP-Seq v1.4.2. IGV tools v2.4.5 was used to visualize the ChIP-Seq tracks. The total H3K27ac ChIP-seq signal is expressed in units of RPM per bin. Homer was used to merge and stitch the peaks within 12.5 kb.

### ChIP-qPCR

ChIP was performed with the Simple Chip Enzymatic Chromatin IP Kit (Cell Signaling) according to the manufacturer’s instructions. The primers for qPCR are listed in Supplementary Table 3.

### ADP/ATP ratio

The ADP/ATP ratio was measured with a commercial ADP/ATP kit purchased from Sigma-Aldrich (Darmstadt, Germany) according to the manufacturer’s instructions. In brief, the cells were incubated with ATP reagent for 1 min, and then luminescence was read for the ATP assay. Then, the cells were incubated for an additional 10 min, and luminescence was read as the background before measuring ADP. Finally, ADP reagent was added and incubated for 1 min, and luminescence was read to calculate the ADP/ATP ratio according to the manufacturer’s instructions.

### Cell viability and colony formation assay

A total of 1000–1500 cells per well were seeded in 96-well plates. Next, cell viability was assessed with Cell Counting Kit-8 (CCK-8) according to the protocol. For colony formation assays, 500–1000 cells per well were seeded in six-well plates and cultured for 2 weeks. Then, colonies were fixed with 100% methanol for 20 min and stained with 0.5% crystal violet for 15 min.

### Cell apoptosis analysis

Cells were collected with trypsinization and washed with phosphate-buffered saline (PBS). Cell death was detected by an Annexin V apoptosis detection kit (R&D Systems, USA). In brief, 1 × 10^5^ cells were incubated with Annexin V-APC and PI for 15 min at room temperature in the dark. Stained cells were measured with a FACSCalibur flow cytometer (BD Biosciences). The data were analyzed by FlowJo software (version 10.0.7).

### Measurement of LDHA activity

To detect intracellular LDHA activity, an LDHA assay kit from Sigma-Aldrich was used according to the manufacturer’s protocols. The LDHA of the samples was calculated using a standard LDHA calibration curve.

### Quantification of RFP-LC3 puncta formation

Cells expressing RFP-LC3 were treated with LDHA knockdown or FX11. Then cells grown on coverslips in a 24-well plates and fixed with 4% paraformaldehyde for 20 min at room temperature and then washed with PBS. Slides were mounted with Fluoromount-G™ Mounting Medium (Them Fisher) and examined by fluorescence microscopy. The percentage of cells with punctate RFP-LC3 fluorescence was calculated by counting the number of the cells with punctate RFP-LC3 fluorescence in RFP-positive cells.

### Animal studies

All of the animal studies were approved by the Ethics Committee of the Tianjin Medical University Cancer Institute and Hospital and were carried out in accordance with the National Institutes of Health Guide for the Care and Use of Laboratory Animals. A total of 2 × 10^6^ control or stable LDHA knockdown B-CPAP cells were injected into 5-week-old female BALB/c nude mice via the tail vein (each group, *n* = 5). To monitor metastatic tumor growth, an IVIS (in vivo imaging system) spectrum (Caliper Life Science, USA) was used to detect the fluorescence intensity of green fluorescent protein every 5 days. Approximately 12 weeks later, the mice were killed, and the lungs and livers were collected and analyzed by hematoxylin and eosin (H&E) staining.

To establish a xenograft tumor model, suspensions of 2 × 10^6^ control or stable LDHA knockdown B-CPAP cells were injected into 5-week-old female NSG mice (each group, *n* = 5). Tumors were measured every 3 days. The tumor volume was calculated using the formula *V* = 0.5 × larger diameter × (smaller diameter)^2^. Suspensions of 2 × 10^6^ B-CPAP cells were injected into 5-week-old female NSG mice and the mice were randomly divided into four groups (each group, *n* = 5). Nine days later, the mice were treated with 2% DMSO, FX11 (3 mg/kg), HCQ (60 mg/kg), and FX11 combined with HCQ once daily for 3 weeks. Tumors were measured every three days. All of the mice were purchased from SPF Biotechnology (Beijing, China).

### Statistical analysis

All data were analyzed with Statistical Product and Service Solutions Ver. 25.0 (IBM, USA) and GraphPad Prism Ver. 7.0 (CA, USA). The data are presented as the mean ± SD of at least three independent experiments. The Chi-square test and multivariate Cox regression analyses were used to estimate the significance of the clinicopathological characteristics of the patient tissue microarray samples. Kaplan–Meier analysis was performed to evaluate survival curves. All data were analyzed with Student’s *t* test (***p < 0.001, **p < 0.01, *p < 0.05).

## Results

### Bioinformatics analysis identifies LDHA as a candidate target gene for PTCs

**Fig. 1:**
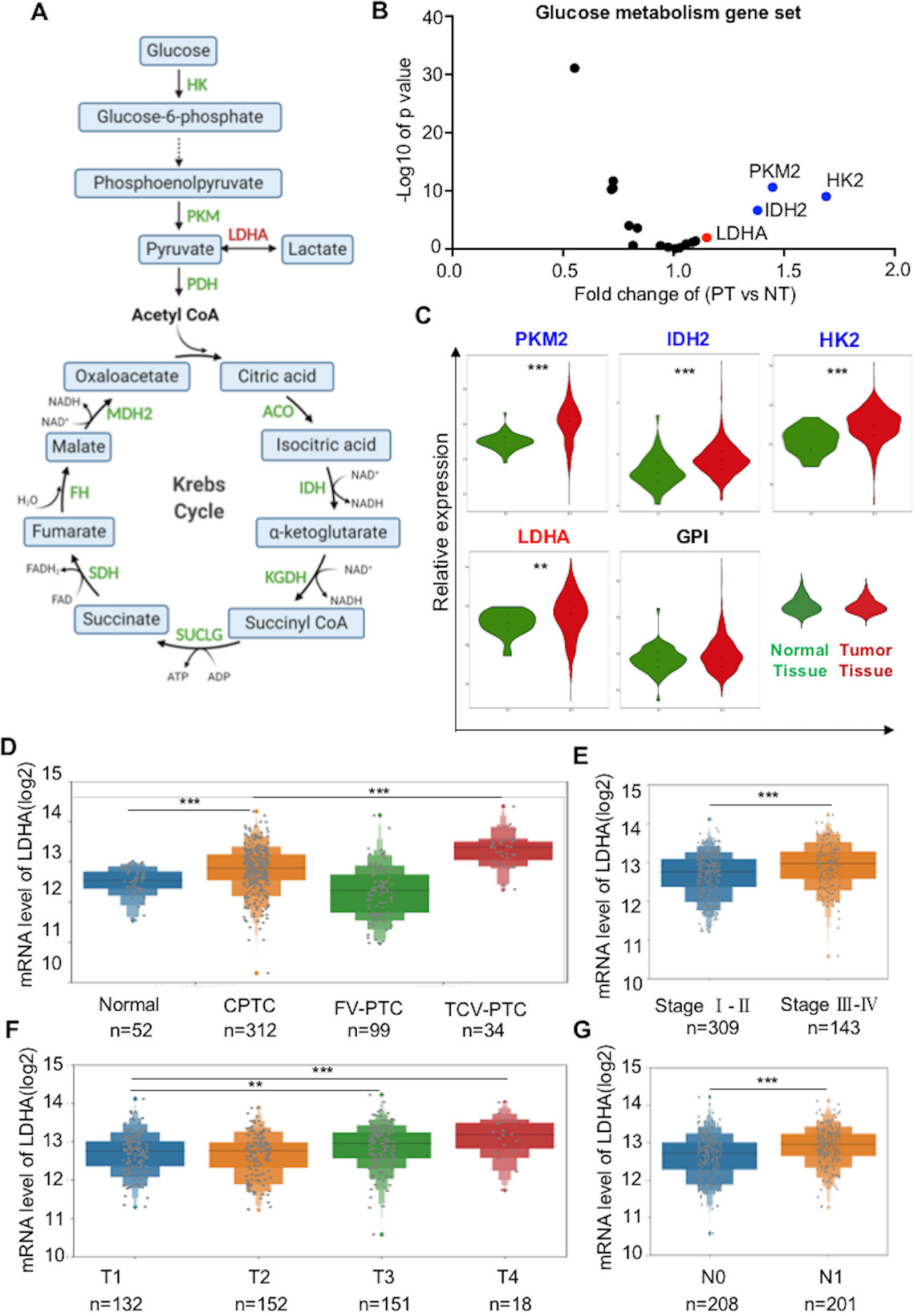
Bioinformatics analysis of enzymes involved in glucose metabolism and identifies LDHA as a candidate target for PTCs. **A** Simplified scheme of glucose metabolism. **B** Fold change of glucose metabolic genes in primary tumor tissue versu*s* normal tissue. **C** Representative glucose metabolic genes that were upregulated in primary PTC tumors. **D** LDHA expression was higher in CPTC and TCV-PTC tissues than in normal tissues. **E** LDHA expression levels of patients in stage III/IV PTC were higher than those in stage I/II. **F** Patients in stage T3 and T4 had higher LDHA expression levels than those in stage T1 and T2. **G** Patients with lymph node metastasis (N1) had higher LDHA levels than those without lymph node metastasis (N0). All *p < 0.05, **p < 0.01, ***p < 0.001.

To explore the potential targets of glucose metabolic enzymes in PTC (Fig. 1A), we first analyzed glucose metabolic gene expression data in PTCs from The Cancer Genome Atlas (TCGA) database. In total, 52 normal tissues and 486 PTCs with clinical medical records were included in our research. The analysis revealed the global view of the relative expression of glucose metabolism genes in primary tumors versus normal tissues (Fig. 1B). PKM2, IDH2, HK2, and LDHA were significantly higher in PTCs, whereas HK1, SUCLG1, and others were higher in normal tissues (Fig. 1C and Supplementary Fig 1A). Among them, we mainly focused on those increased in PTCs because their inhibitors have already been established. Based on the preferential use of lactate rather than glucose is a hallmark of many kinds of cancer, but the function of LDHA is still elusive. Therefore, in this research, we focused specifically on the effect and mechanism of LDHA in PTCs.

Analysis of the TCGA database showed that LDHA expression levels were much higher in classic PTCs (CPTCs) than in normal tissues, and the tall cell variant PTCs, which are known to have a more aggressive clinical behavior, had a higher LDHA expression level than CPTCs (Fig. 1D, *p* < 0.001). In addition, LDHA expression levels were significantly higher in patients in stage III/IV than those in stage I/II (Fig. 1E, *p* < 0.001). Patients in the T3 and T4 groups had higher LDHA expression levels than those in the T1 and T2 groups (Fig. 1F, *p* < 0.001). Moreover, LDHA expression levels were significantly higher in patients with lymph node metastasis (N1 group) than those without lymph node metastasis (N0 group) (Fig. 1G, *p* < 0.001). We also found that LDHA expression levels were much higher in patients with extrathyroidal extension than those without extrathyroidal extension (Supplementary Fig 1B, *p* < 0.001). Overall, these results revealed that LDHA is highly expressed in PTC patients, especially in those with later stages of the disease.

### High expression levels of LDHA are associated with aggressive clinicopathological features and poor prognosis

**Fig. 2:**
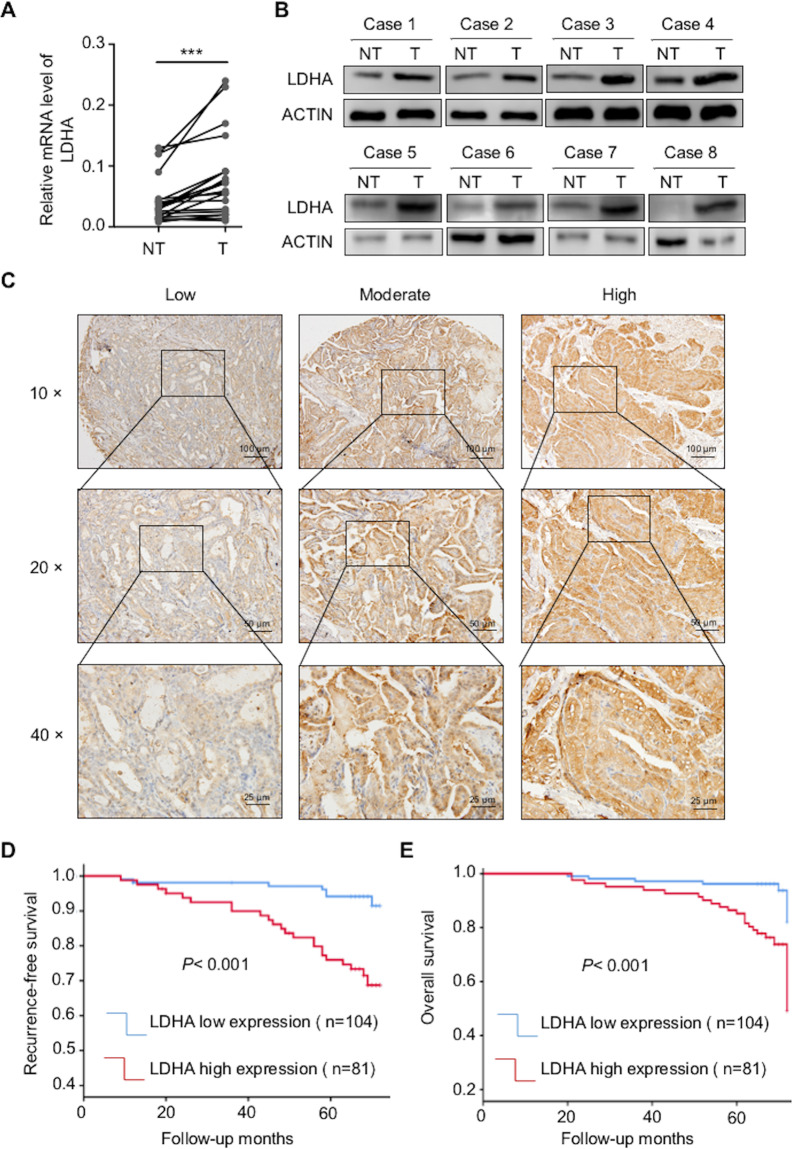
The expression of LDHA in PTCs and Kaplan–Meier estimates of the probability of OS and RFS in PTC patients. **A** Expression levels of LDHA mRNA in 20 paired normal tissues and primary tumor tissues. **B** Expression levels of LDHA in eight paired normal tissues and primary tumor tissues. **C** LDHA was found to be mainly located in the cytoplasm of PTCs. Representative IHC images of weak staining, moderate staining, and strong staining were shown. **D**, **E** Kaplan–Meier survival analysis showed that high LDHA levels were significantly associated with poor RFS and OS in PTC patients. All *p < 0.05, **p < 0.01, ***p < 0.001.

Next, we determined the expression of LDHA at both the mRNA and protein levels and found that they were much higher in PTC tissues than in paired normal tissues (Fig. 2A, B). The densitometric analysis of western blots is shown in Supplementary Fig 2A. To further explore the correlation between the expression of LDHA and clinicopathological features, 185 human specimens were subjected to immunohistochemical analysis (Fig. 2C). We found that LDHA was mainly located in the cytoplasm of PTCs. Univariate analysis showed that T stage, N stage, and TNM stage were significantly correlated with high LDHA levels (*p* < 0.05) (Supplementary Table 1).

The median follow-up of the 185 patients was 65 months (range from 9 to 72 months). Among these patients, 30 experienced recurrence or persistent disease, and 21 died of cancer-related death or other reasons. Several factors, such as age, sex, TNM stage, multifocality, and LDHA expression level were included in the Cox proportional hazards model. The multivariate COX regression analysis showed that TNM stage (hazard ratio (HR) = 4.950, 95% confidence interval (CI) = 1.299–18.864, *p* = 0.019) and LDHA expression level (HR = 4.848, 95% CI = 1.890–12.44, *p* = 0.01) were independent factors of recurrence-free survival (RFS). In addition, TNM stage (HR = 5.414, 95% CI = 1.389–21.096, *p* = 0.015) and LDHA expression level (HR = 5.545, 95% CI = 2.026–15.175, *p* = 0.001) were independent factors of OS (Supplementary Table 2).

As the Kaplan–Meier curves showed in Fig. 2D, E, patients with higher LDHA expression levels had shorter RFS and OS than patients with lower LDHA expression levels. Overall, patients with high LDHA expression have a poor prognosis, thus indicating that LDHA is a potential prognostic marker for PTCs.

### LDHA promotes the migration and invasion of PTC cells in vitro and in vivo

**Fig. 3:**
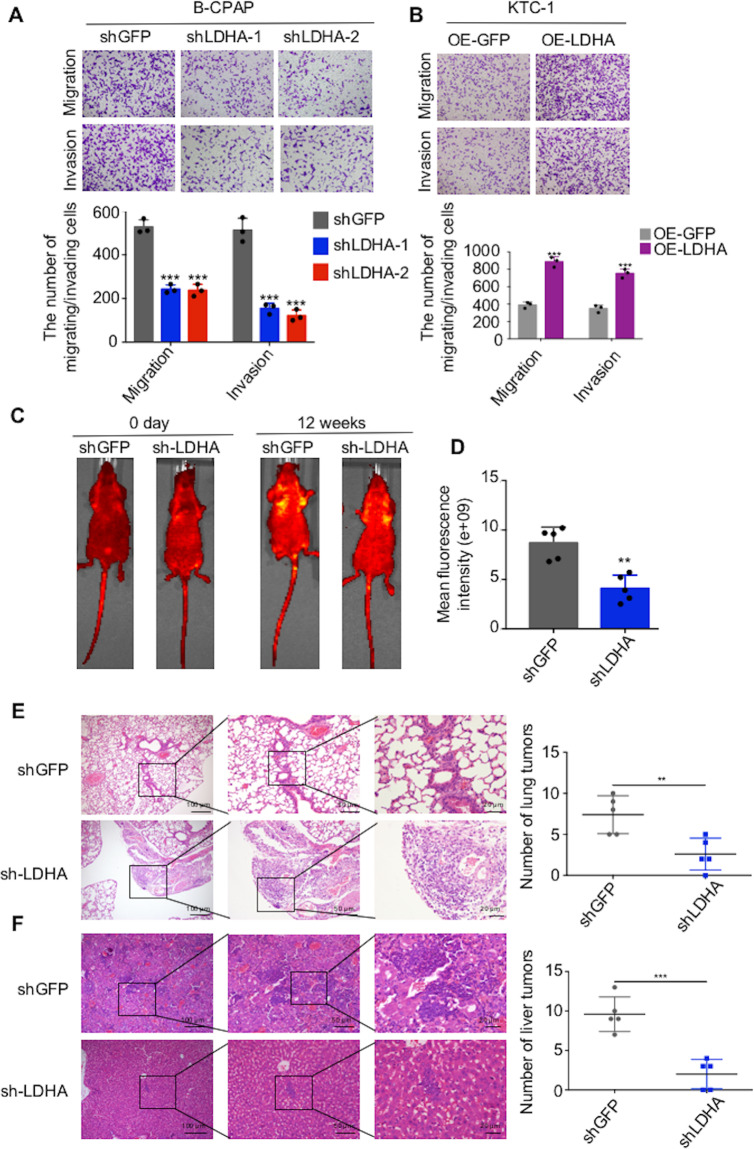
LDHA promotes the migration and invasion of PTC cells in vitro and in vivo. **A** B-CPAP cells were transfected with two different LDHA knockdown plasmids. Representative images of migration/invasion are shown. **B** Representative images of migrating/invading KTC-1 cells expressing LDHA. **C** In vivo bioluminescence images of B-CPAP cells transfected with the LDHA knockdown plasmid. **D** The mean fluorescence intensity of the LDHA knockdown group was significantly lower than that of the control group. **E** Representative images of lung metastatic foci. The number of lung metastatic foci was significantly lower in LDHA knockdown group than in control group. **F** Representative images of liver metastatic foci. The number of liver metastatic foci was significantly lower in LDHA knockdown group than in control group. The data are presented as the mean ± SD. All *p < 0.05, **p < 0.01, ***p < 0.001.

Poor prognosis is closely related to metastasis, and the high LDHA level has been shown to be correlated with lymph node metastasis, as mentioned above. Thus, we investigated whether LDHA affected the migration or invasion of PTC cells. We detected the expression of LDHA in different cell lines by western blot and qPCR assays. Compared with those in Nthy-ori 3–1 cells, the protein and mRNA levels of LDHA were higher in B-CPAP and TPC-1 cells and lower in KTC-1 cells (Supplementary Fig 3A, B). Then, we knocked down LDHA in B-CPAP and TPC-1 cells and found that the migration and invasion abilities of these cells were significantly decreased (Fig. 3A, Supplementary Fig 3C, D). Moreover, the ectopic expression of LDHA promoted the migration and invasion of KTC-1 cells (Fig. 3B and Supplementary Fig 3D). Western blot and qPCR assays were used to confirm the efficiencies of the knockdown and overexpression of LDHA (Supplementary Fig. 3E, F).

Furthermore, to explore the effect of LDHA in vivo, we established a lung metastasis model by directly injecting B-CPAP cells into the tail veins of mice (Fig. 3C). The IVIS images showed that the mean fluorescence intensity of the LDHA knockdown group was significantly lower than that of the control group (Fig. 3D). Additional H&E staining of the isolated lung and liver tissues confirmed that there were significantly fewer metastases in the LDHA knockdown group than in the control group (Fig. 3E, F). In brief, both the in vitro and in vivo results demonstrated the roles of LDHA in promoting PTC invasion and metastasis.

### LDHA promotes the proliferation and inhibits the apoptosis of PTC cells in vitro and in vivo

**Fig. 4:**
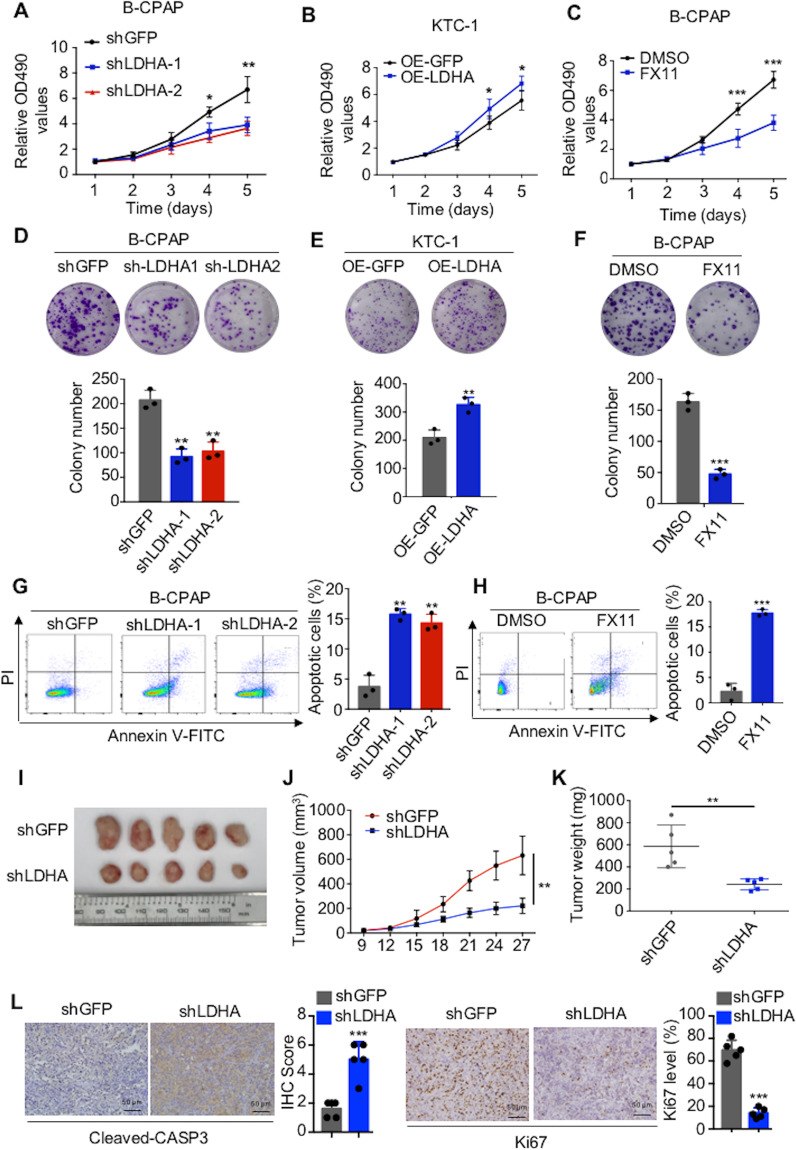
LDHA promotes the proliferation and inhibits the apoptosis of PTC cells in vitro and in vivo. **A**–**C** CCK-8 assays and **D**–**F** colony formation assays showed that LDHA knockdown or inhibition significantly suppressed cell proliferation in B-CPAP cells and that the overexpression of LDHA promoted cell proliferation in KTC-1 cells. **G**, **H** Flow cytometry indicated that LDHA knockdown or inhibition significantly induced cell apoptosis in B-CPAP cells. **I** Five representative tumors from LDHA knockdown cells are shown. Tumor volumes **J** are presented with growth curves, and tumor weights **K** were measured. **L** Representative IHC images of cleaved caspase 3 expression and Ki-67 staining in xenografted tumor specimens of control and LDHA knockdown mice are shown. The data are presented as the mean ± SD. All *p < 0.05, **p < 0.01, ***p < 0.001.

In addition to metastasis, tumorigenesis is also an important risk factor for poor prognosis. CCK-8 assays (Fig. 4A and Supplementary Fig 4A) and colony formation assays (Fig. 4D and Supplementary Fig 4B) revealed that LDHA knockdown in B-CPAP and TPC-1 cells significantly inhibited cell proliferation, whereas overexpression of LDHA in KTC-1 cells significantly promoted cell proliferation (Fig. 4B and E). Next, we applied the LDHA inhibitor FX11 to PTC cells, which significantly inhibited the activity of LDHA (Supplementary Fig 4C, D). CCK-8 (Fig. 4C and Supplementary 4E) and colony formation (Fig. 4F and Supplementary Fig 4F) assays showed that LDHA inhibition significantly inhibited cell proliferation. Furthermore, we examined the effect of LDHA on cell apoptosis by flow cytometry. LDHA knockdown and FX11-induced cell apoptosis (Fig. 4G, H and Supplementary Fig 4A, G, H).

Moreover, to evaluate the effect of LDHA on tumorigenesis in vivo, we generated xenografts with B-CPAP cells stably knocking down LDHA. Compared with those of control group tumors, the volumes and weights of LDHA knockdown tumors were significantly decreased (Fig. 4I–K). In addition, xenografts isolated from LDHA knockdown mice showed significantly more apoptotic cells and fewer proliferating cells, as measured by cleaved caspase 3 and Ki-67 with IHC staining, respectively (Fig. 4L). Overall, LDHA played an important tumorigenic role in promoting PTC cell growth in vitro and in vivo.

### LDHA promotes EMT and sustains the normal mitochondrial function of PTCs

**Fig. 5:**
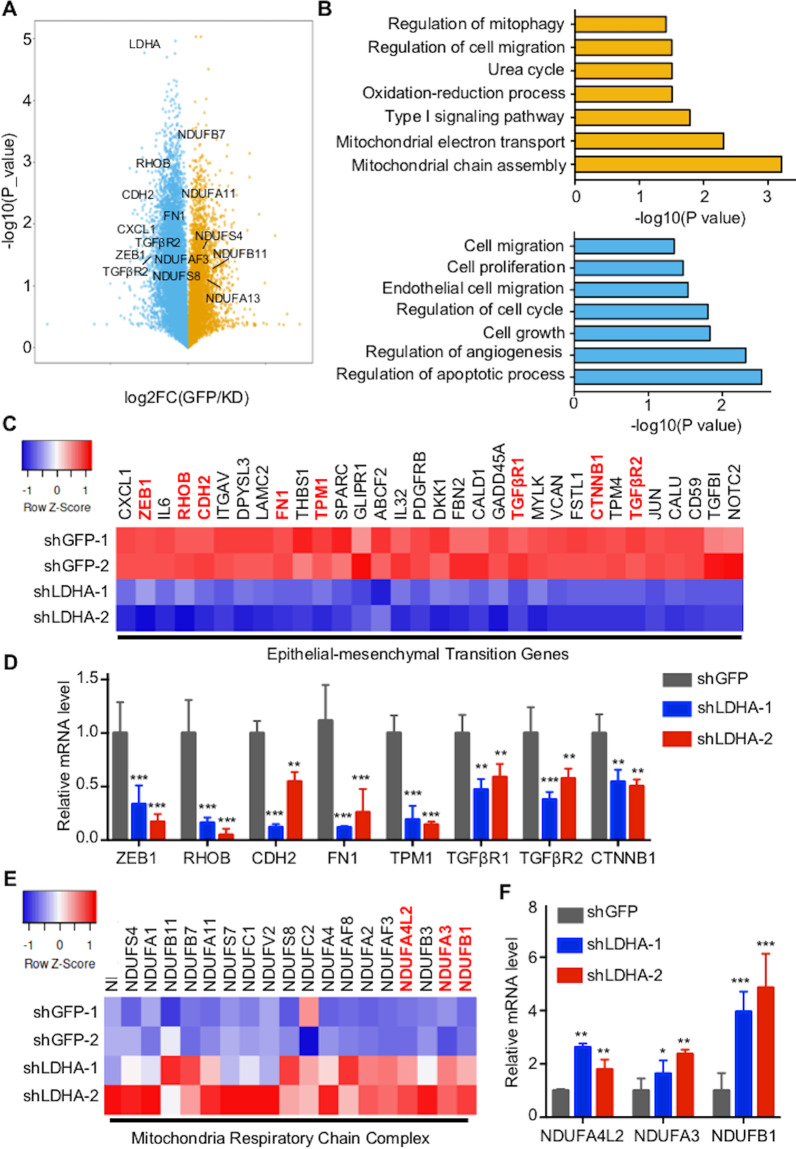
Discovery of LDHA regulated genes in PTC. **A** Volcano plot of the gene expression changes that occurred after LDHA knockdown in B-CPAP cells assayed by RNA-Seq. **B** Summary of the significantly enriched pathways in B-CPAP cells after LDHA knockdown. **C** Heatmap from the RNA-Seq data showing the differentially expressed genes involved in the EMT process. **D** qPCR analysis of representative genes related to the EMT process after LDHA knockdown. The expression data were normalized to that of Actin in GFP knockdown cells. **E** Heatmap of the RNA-Seq data show that the differentially expressed genes were related to the mitochondrial respiratory chain complex. **F** qPCR analysis of representative genes related to the mitochondrial respiratory chain complex after LDHA knockdown. All *p < 0.05, **p < 0.01, ***p < 0.001.

To assess the function of LDHA in PTCs, we performed transcriptome analysis through high-throughput RNA-Seq of control and LDHA knockdown cells. Transcriptome analysis revealed that 283 genes were upregulated, whereas 1556 genes were downregulated, including ZEB1, CDH2, and TGFβR1 (Fig. 5A). Global pathway analysis revealed that mitochondrial chain assembly, cell migration, and cell mitophagy were significantly enriched in the upregulated genes, whereas apoptotic process, cell growth, and cell migration were significantly enriched in the downregulated genes (Fig. 5B). Further analysis of the RNA-Seq data showed that the downregulated genes were involved in the EMT process and the upregulated genes were related to the mitochondrial respiratory chain complex (Fig. 5C, E). The representative genes were confirmed by qPCR (Fig. 5D, F). In summary, these results revealed that LDHA plays critical roles in the EMT process and maintains the normal function of mitochondria in PTCs.

### LDHA promotes the transcription of genes involved in the EMT process

**Fig. 6:**
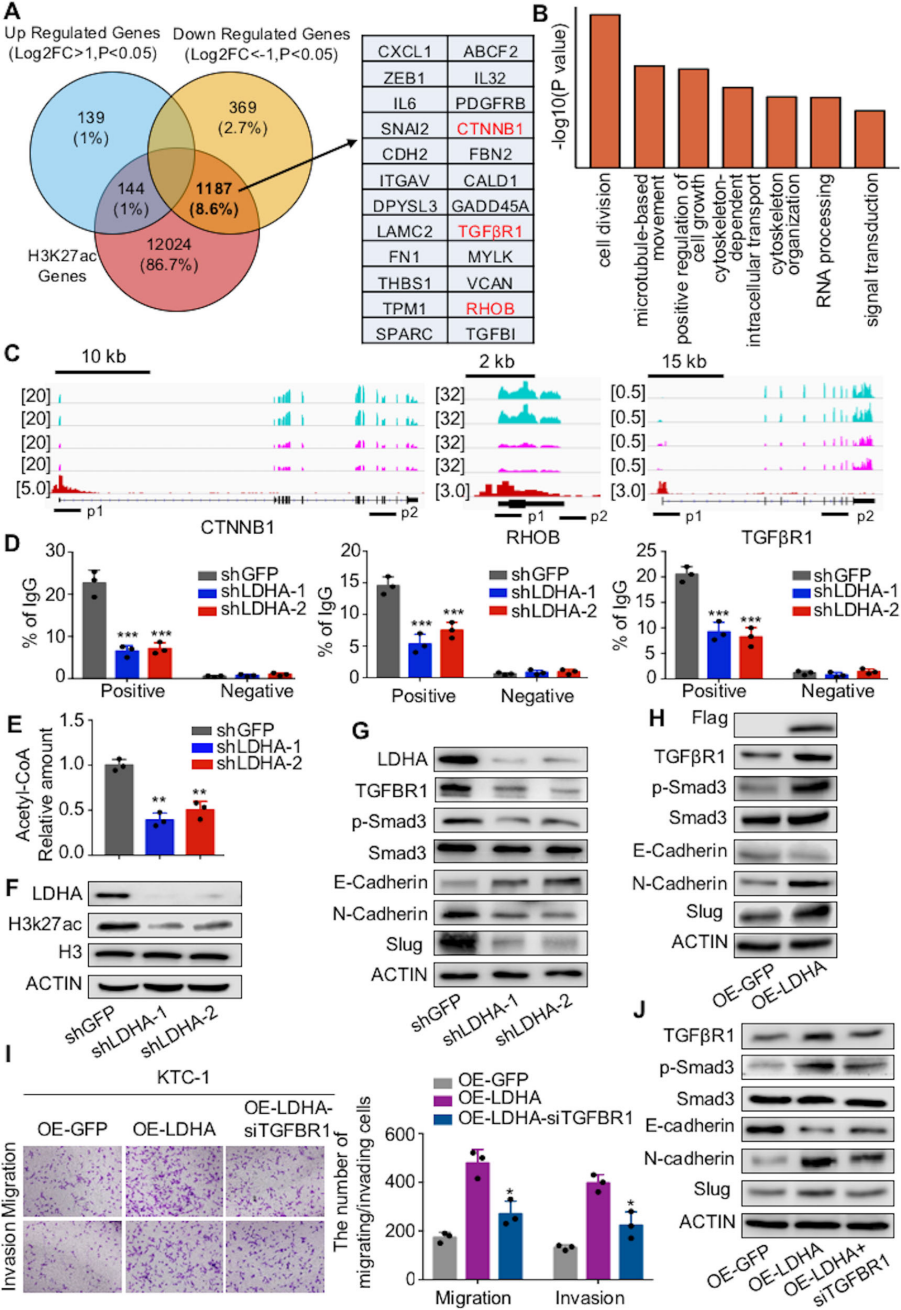
LDHA promotes the transcription of genes involved in the EMT process. **A** Venn diagram illustrating the overlap between genes from the RNA-Seq data after LDHA knockdown and genes from the ChIP-Seq data of H3K27ac modification in B-CPAP cells. **B** Summary of the significantly enriched pathways of the downregulated genes bound by H3K27ac. **C** RNA-Seq peaks of CTNNB1, RHOB, and TGFβR1 treated with LDHA knockdown, as well as H3K27ac binding peaks, are shown. **D** ChIP-qPCR was performed with B-CPAP cells using H3K27ac and IgG antibodies to determine the expression levels of CTNNB1, RHOB, and TGFβR1. **E** Intracellular acetyl-CoA levels were measured in B-CPAP cells treated with LDHA knockdown. **F** H3K27ac levels were decreased after LDHA knockdown in B-CPAP cells. **G**, **H** Western blot assays showed the changes in TGFβR1, phosphorylated Smad3, E-cadherin, N-cadherin, and Slug in B-CPAP cells with LDHA knockdown and in KTC-1 cells expressing LDHA. **I** Transwell assays indicated that the knockdown of TGFβR1 partially reversed the increased migration and invasion ability of KTC-1 cells expressing LDHA. The data are presented as the mean ± SD. **J** KTC-1 cells stably expressing LDHA were infected with siRNA targeting TGFβR1. Western blotting was used to detect the expression levels of TGFβR1 and the phosphorylation levels of Smad3, E-cadherin, N-cadherin, and Slug. All *p < 0.05, **p < 0.01, ***p < 0.001.

Then, the molecular mechanism by which LDHA knockdown inhibits the migration and invasion of PTCs was determined. Recently, it has been demonstrated that H3K27ac marks active promoters and enhancers and is thus an indicator of gene expression^36^. Previously, we performed a ChIP-Seq analysis of H3K27ac modification in PTC^23^. An integrative analysis of transcriptome data and ChIP-Seq H3K27ac modification data was performed. Approximately 76.1% of downregulated genes overlapped with the ChIP-Seq H3K27ac data (Fig. 6A). GO analysis showed that these genes were mainly enriched in cell division, microtubule-based movement, and regulation of cell growth (Fig. 6B). EMT-relative genes, including CTNNB1, RHOB, and TGFβR1 had strong H3K27ac signals (Fig. 6C). Then, we verified the representative genes through ChIP-qPCR of H2K27ac and found that LDHA knockdown significantly decreased the transcription levels of CTNNB1, RHOB, and TGFβR1 (Fig. 6D).

Decreased histone acetylation may be due to the low intracellular acetyl-CoA level, which links metabolism with chromatin remodeling^25,26^. Because the production of acetyl-CoA relies on normal mitochondrial function, we measured the intracellular acetyl-CoA levels in LDHA knockdown cells. LDHA knockdown significantly decreased the intracellular acetyl-CoA level (Fig. 6E and Supplementary Fig 5A). The total H3K27ac level decreased after LDHA knockdown (Fig. 6F and Supplementary Fig 5B). In addition to H3K27ac, we also detected the levels of other epigenetic markers, including H3K27me3 and H3K36me3. We did not observe changes after LDHA knockdown (Supplementary Fig 5C), while ChIP-qPCR analysis of H3K27me3 and H3K36me3 showed that the transcription levels of CTNNB1, RHOB, and TGFβR1 were significantly decreased after LDHA knockdown (Supplementary Fig 5D, E). Furthermore, western blot analysis revealed that the epithelial marker E-cadherin was negatively regulated by LDHA, while TGFβR1, the EMT-related transcription factor Slug and mesenchymal markers, including N-cadherin and vimentin, were positively regulated (Fig. 6G, H and Supplementary Fig 5F).

To investigate whether LDHA regulates the EMT process through TGFβR1 signaling, we transiently transfected KTC-1 cells with siRNA to inhibit the transcription of TGFβR1 (Supplementary Fig 5G). Our results showed that TGFβR1 knockdown abolished the positive effects of LDHA on cell migration and invasion (Fig. 6I). Additionally, the increased expression of E-cadherin and decreased expression of Slug, N-cadherin, and vimentin were partly blocked by TGFβR1 knockdown (Fig. 6J). Taken together, these results indicated that LDHA induced EMT-related genes transcription to promote the migration and invasion of PTC cells.

### LDHA regulates tumorigenesis and autophagy through the AMPK signaling pathway

**Fig. 7:**
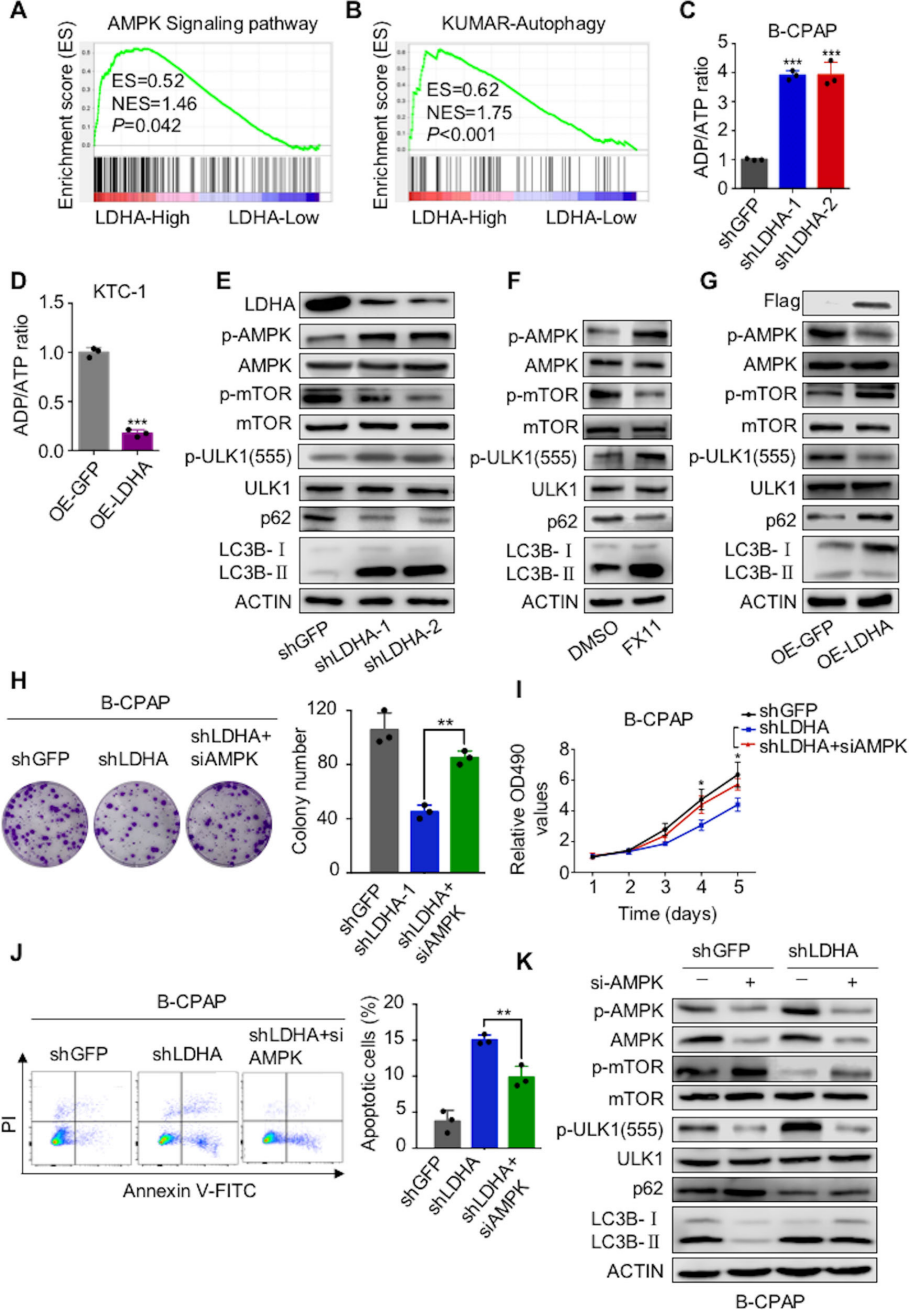
LDHA regulates tumorigenesis and autophagy through the AMPK signaling pathway. **A**, **B** Gene expression data acquired from the TCGA database were subjected to GSEA v2.2.0; the results showed that LDHA expression was correlated with AMPK signaling and autophagy-related pathways. **C**, **D** The ADP/ATP ratio assay showed that LDHA knockdown increased the ADP/ATP ratio in B-CPAP cells, whereas overexpression of LDHA decreased the ADP/ATP ratio in KTC-1 cells. **E**–**G** Western blotting was used to detect phosphorylated AMPK, phosphorylated mTOR, phosphorylated ULK1, p62, and LC3BII/I in B-CPAP cells treated with LDHA knockdown or FX11 (10 μM for 24 h) and in KTC-1 cells treated with LDHA expressing. Colony formation assay **H** and CCK-8 assay **I** showed that AMPK knockdown partially attenuated the inhibited proliferation induced by LDHA knockdown in B-CPAP cells. **J** Flow cytometry indicated that AMPK knockdown partially reversed the apoptosis induced by LDHA knockdown in B-CPAP cells. **K** B-CPAP cells stably knocking down LDHA were infected with siRNAs targeting AMPK. Western blotting was used to detect phosphorylated AMPK, phosphorylated mTOR, phosphorylated ULK1, p62, and LC3BII/I expression levels. The data are presented as the mean ± SD. All *p < 0.05, **p < 0.01, ***p < 0.001.

GSEA of data from the TCGA database showed that LDHA was correlated with AMPK signaling, autophagy, and apoptosis in PTCs (Fig. 7A, B and Supplementary Fig 6A). As a metabolic checkpoint, AMPK is involved in the tumorigenesis and progression of many kinds of cancers^30^. Thus, we further explored whether the AMPK signaling pathway plays an important role in the molecular regulation by which LDHA alters PTC cell tumorigenesis.

First, we examined the ADP/ATP ratio, which is known to signal AMPK signaling, and found that LDHA knockdown increased the ADP/ATP ratio, while ectopic LDHA expression decreased the ratio (Fig. 7C, D and Supplementary Fig 6B). Furthermore, western blot analysis showed that when LDHA was knocked down or inhibited, the phosphorylated AMPK level was increased (Fig. 7E, F and Supplementary Fig 6C, D). As the key downstream target of AMPK signaling, mTOR is involved in cell growth, cell proliferation, and cell survival processes^37^. LDHA knockdown or inhibition decreased the phosphorylation level of mTOR. ULK1 is another node at which AMPK regulates a specific cellular process, autophagy^30^. Western blot analysis demonstrated that when LDHA was knocked down or inhibited, the ULK1 phosphorylation level increased and autophagy was activated, which was indicated by the decrease in p62 and the increase in LC3BII/I (Fig. 7E, F and Supplementary Fig 6C, D). To further validate the activation of autophagy, we overexpressed RFP-LC3 in PTC cells and found that LDHA knockdown or inhibition significantly increased LC3 puncta (Supplementary Fig 6E, F). At the same time, ectopic LDHA expression inhibited AMPK signaling, which was indicated by the upregulation of the phosphorylated level of mTOR and the downregulation of the phosphorylated level of ULK1. In addition, the overexpression of LDHA led to the inhibition of autophagy, which was indicated by the increase in p62 and the decrease in LC3BII/I (Fig. 7G).

Furthermore, we investigated the function of LDHA mediated by AMPK signaling. Colony formation (Fig. 7H and Supplementary Fig 6G) and CCK-8 (Fig. 7I and Supplementary Fig 6H) assays showed that the knockdown of AMPK significantly reversed the positive effects of LDHA on cell proliferation. Flow cytometry showed that AMPK knockdown abolished the inhibitory effects of LDHA on cell apoptosis (Fig. 7J and Supplementary Fig 6I). In addition, the downregulation of phosphorylated mTOR and the upregulation of phosphorylated ULK1 were partly rescued by incubation with AMPK siRNA. Meanwhile, the activation of autophagy induced by LDHA knockdown was partly blocked by AMPK knockdown (Fig. 7K and Supplementary Fig 6J).

### The combination of FX11 and HCQ synergistically inhibits tumorigenesis in vitro and in vivo

**Fig. 8:**
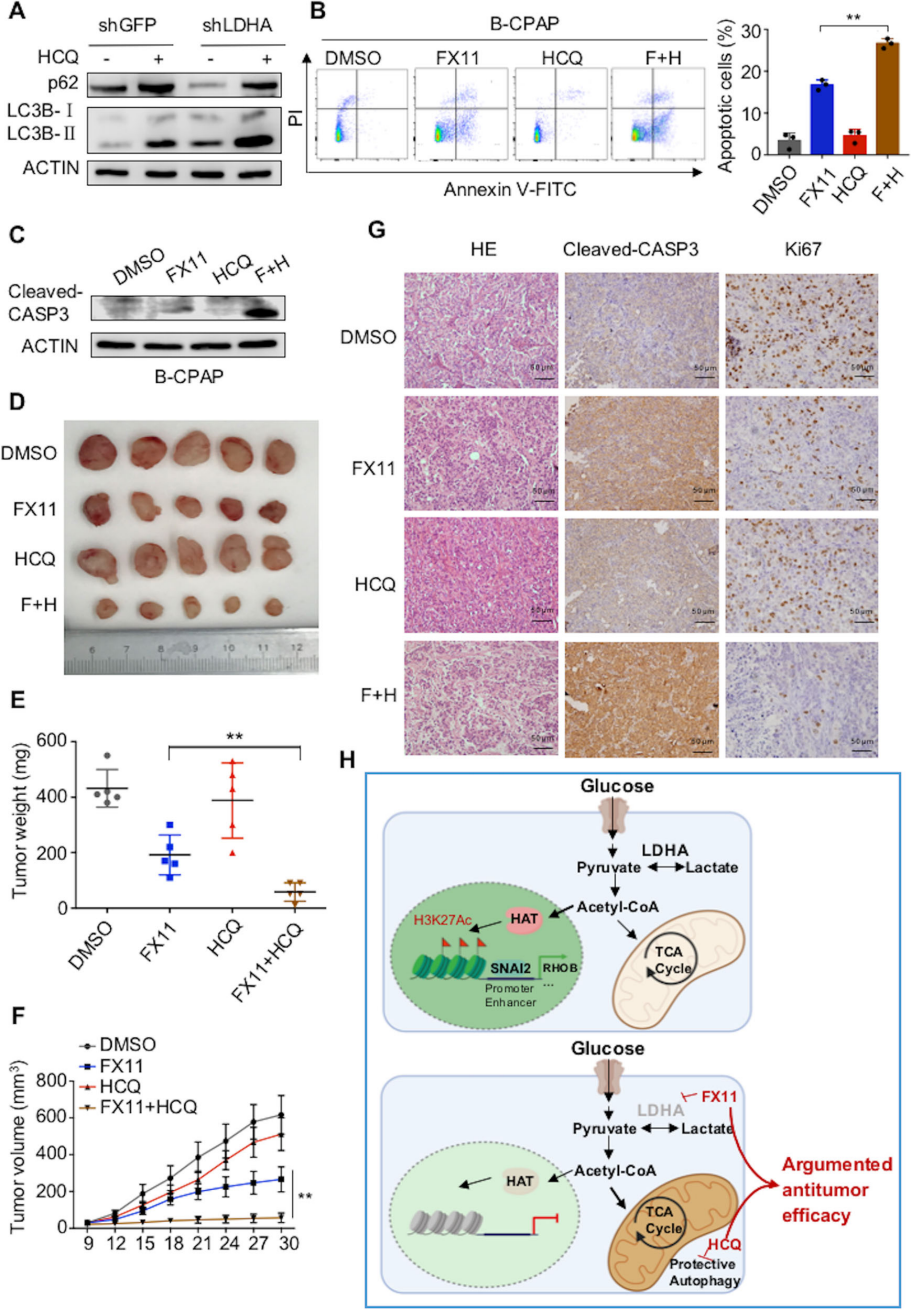
HCQ enhanced the antitumor effect of FX11 in vitro and in vivo. **A** B-CPAP cells with stable LDHA knockdown were treated with the autophagy inhibitor hydroxychloroquine (HCQ). Western blotting was used to detect p62 and LC3BII/I levels. **B** Apoptosis of B-CPAP cells exposed to 10 μM of FX11 for 24 h with/without HCQ (10 μM for 24 h) was detected by flow cytometry. The data are expressed as the mean ± SD. **C** B-CPAP cells were exposed to FX11 (10 μM for 24 h) with/without HCQ (10 μM for 24 h), and western blotting was used to detect cleaved caspase 3 levels. **D** FX11 (3 mg/kg) with/without HCQ (60 mg/kg) was given to mice bearing papillary thyroid tumors for 21 days. Tumor weights were measured on day 30 **E**, and tumor volumes **F** are presented with growth curves. The data are presented as the mean ± SD. **G** H&E staining and representative IHC images of cleaved caspase 3 expression and Ki-67 staining of xenografted tumor specimens from the FX11 with/without HCQ groups are shown. All *p < 0.05, **p < 0.01, ***p < 0.001.

Western blot analysis showed that HCQ inhibited the autophagy induced by LDHA knockdown (Fig. 8A and Supplementary Fig 7A). Autophagy is activated by LDHA knockdown and plays protumor and antitumor roles in cancers^38^. Then, we combined the autophagy inhibitor HCQ, which inhibits autophagy by preventing lysosomes from degrading and recycling the materials engulfed in the autophagosome, with FX11 in PTCs. We found that FX11 and HCQ alone could induce cell apoptosis to some extent, while their combination led to a better response (Fig. 8B and Supplementary Fig 7B). Western blot analysis also indicated that FX11 combined with HCQ significantly upregulated the cleaved caspase 3 levels (Fig. 8C and Supplementary Fig 7C).

Moreover, to determine the effect of the combination of FX11 and HCQ in vivo, 20 female BALB/c nude mice were subcutaneously injected with B-CPAP cells and treated with FX11 and HCQ (Fig. 8D). The results showed that the tumor weights and tumor volumes were significantly inhibited by FX11, while the combination of FX11 and HCQ resulted in greater tumor suppression (Fig. 8E and F). Neither FX11 nor HCQ led to significant weight loss in the mice (Supplementary Fig 7D). IHC staining of tumors showed that the combination of FX11 and HCQ could significantly increase the expression of cleaved caspase 3 and decrease the expression of Ki-67 in vivo (Fig. 8G and Supplementary Fig 7E). Overall, HCQ could significantly enhance the antitumor effect of FX11 in vitro and in vivo.

## Discussion

In this study, we determined that in addition to energy supply, LDHA was also involved in glucose metabolism, maintaining the production of acetyl-CoA. Some acetyl-CoA goes to the TCA cycle to produce ATP, whereas some acetyl-CoA participates in histone 3 lysine 27 acetylation. When LDHA was knocked down or inhibited, the intracellular acetyl-CoA level was decreased, leading to EMT-related gene transcription inhibition. Meanwhile, protective autophagy was activated via AMPK signaling due to the downregulation of LDHA, and the inhibition of autophagy augmented the antitumor effect of FX11 in PTCs (Fig. 8H).

EMT is thought to occur in the process of thyroid cancer invasion and metastasis, especially in PTC^39^. LDHA leads to EMT-like changes to promote cell migration and invasion in various cancers^15,40,41^. In this research, we integrated gene expression data with genomic mapping of the H3K27ac binding profile to assess how alterations in histone acetylation by LDHA knockdown affect the transcriptomic landscape of PTCs. We found that by regulating the intracellular acetyl-CoA level, LDHA gained the H3K27ac mark at the regulatory regions of EMT-related genes. Histone acetylation requires acetyl-CoA as a substrate, with glucose being a critical source. Low acetyl-CoA levels after LDHA knockdown suggest two possibilities: (1) less citrate can be exported from mitochondria for acetyl-CoA regeneration owing to enhanced TCA cycle activity^27^; and (2) LDHA downregulation leads to an impairment in mitochondrial function, which induces an inability of the metabolic pathway to synthesize sufficient acetyl-CoA^42^. However, the detailed molecular events following LDHA and acetyl-CoA merit further research. In addition, the loss of H3K27ac can further recruit more repressive marker (H3K27me3) and evict active marker (H3K36me3) from EMT gene promoters, consequently modulating the transcription of EMT genes. In addition to H3K27ac, recent research also showed that the epigenetic modification of histone lysine residue lactylation could also stimulate gene transcription directly^43^. The relationship between LDHA and the lactylation of EMT-related genes also needs to be further explored.

The exploration of the biological function of LDHA suggested that the inhibition of LDHA suppressed tumor growth in vitro and in vivo, which is consistent with the findings of the previous studies^13,20,44^. In addition, FX11, an inhibitor of LDHA, has been determined to have preclinical efficiency in prostate cancer, osteosarcoma, pancreatic cancer, and lymphoma^17,19,45,46^. Our data also showed that FX11 had an antitumor effect on PTCs in vitro and in vivo. However, in a pancreatic cancer model, research revealed that FX11 as monotherapy had no effect on tumor growth. This was owing to the sensitivity to LDHA inhibition in pancreatic cancer depending on the status of TP53, with TP53 wild-type patients showing complete resistance to FX11 and TP53 mutant patients showing a therapeutic response^47^. As a downstream target of TP53, TIGAR could lower glycolytic flux and reduce the sensitivity of cancers to reactive oxygen species-related apoptosis^48,49^. Of note, the expression of TIGAR was higher in wild-type TP53 but lower in mutant TP53. This may be the potential mechanism by which pancreatic cancer shows different responses to LDHA inhibitors. However, whether the correlation between TIGAR and TP53 exists in PTCs needs to be further investigated.

We observed that autophagy was induced by LDHA inhibition. In recent years, autophagy has been found to play a dual role in the development and progression of tumors^50^. On the one hand, it suppresses tumorigenesis by inducing cancer cell death and inhibiting cell survival^38^. On the other hand, it can protect cells from undergoing programmed cell death, which provides proof for why the inhibition of autophagy could enhance the effect of other agents^31,51,52^. In PTC cells, it showed that vemurafenib not only inhibited the proliferation of cancer cells but also induced a high level of autophagy and the inhibition of autophagy augmented the efficiency of vemurafenib^53^. Similarly, we combined FX11 with HCQ, which is under clinical trials, in PTC cells and found that the inhibition of autophagy augmented the antitumor effect of FX11 in vitro and in vivo. This finding indicated that the autophagy induced by LDHA knockdown may play a protective role in PTC cells.

## Conclusion

In summary, for the first time, our research showed that LDHA is a potential biomarker and a valuable therapeutic target for PTCs. Moreover, FX11 combined with HCQ augmented the antitumor effect in PTCs, which shed light on this combination strategy in the treatment of PTCs.

## Supplementary information

Figure S1

Figure S2

Figure S3

Figure S4

Figure S5

Figure S6-1

Figure S6-2

Figure S7

Supplementary Information

## Data Availability

The data sets used and/or analyzed during the current study are available from the corresponding author on reasonable request.
