# Supplementary figures and images for: LDHA induces EMT gene transcription and regulates autophagy to promote the metastasis and tumorigenesis of papillary thyroid carcinoma
Source: Cell Death Dis. 2021 Apr 1;12(4):347. doi: 10.1038/s41419-021-03641-8 (PMC8017009; doi:10.1038/s41419-021-03641-8)

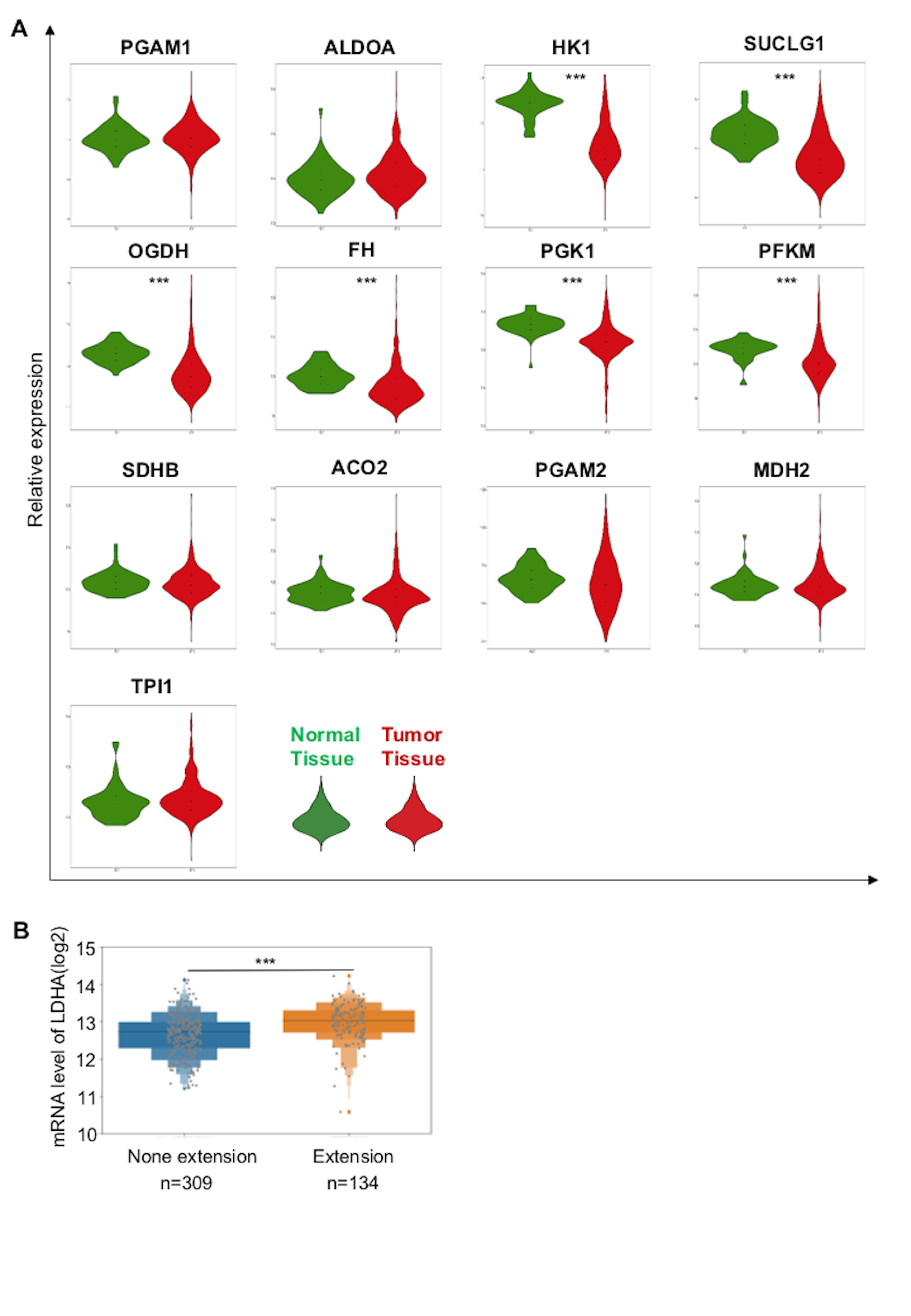

Supplement: Supplementary file 1 — Figure S1 [file 41419_2021_3641_MOESM1_ESM.tif]

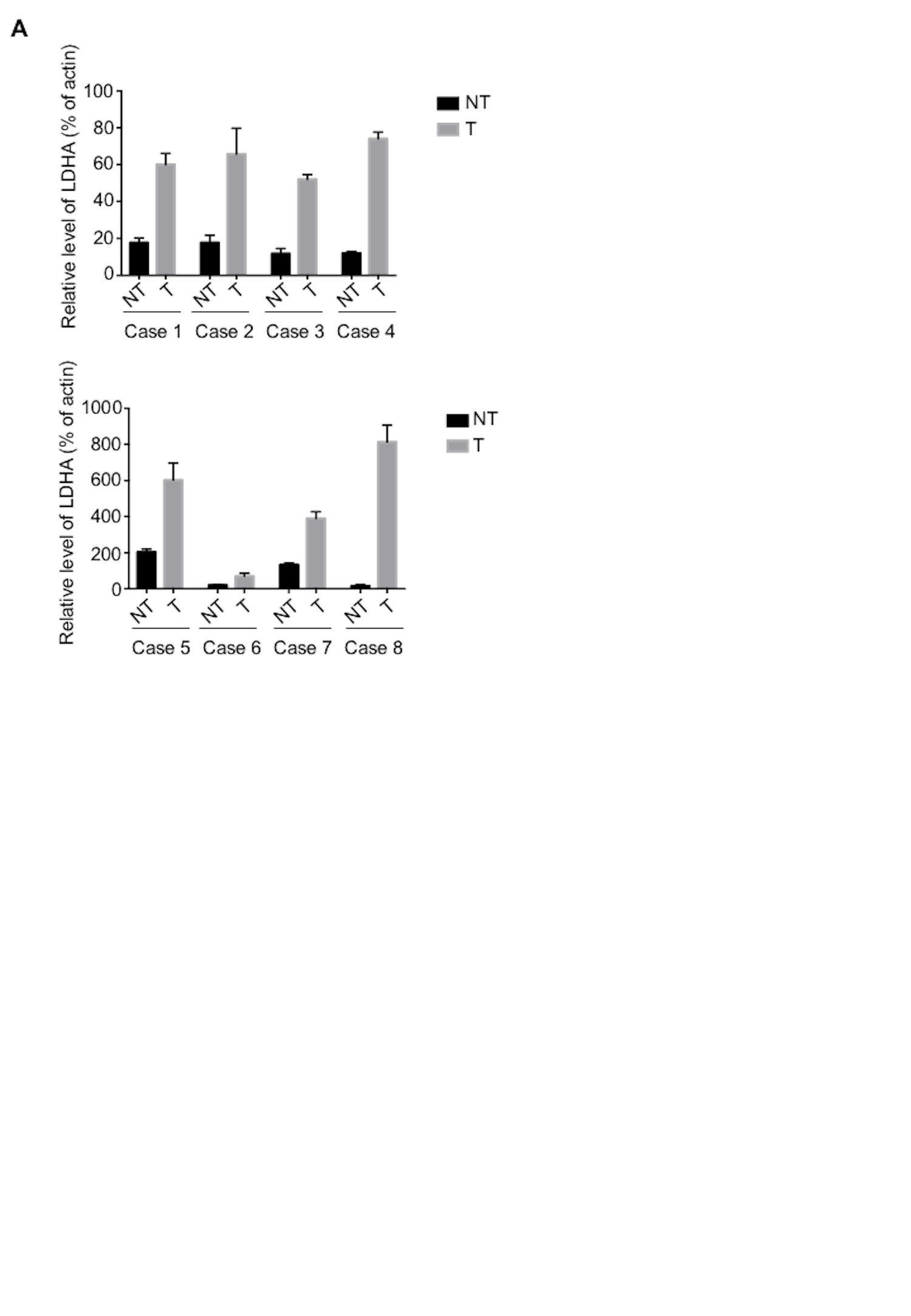

Supplement: Supplementary file 2 — Figure S2 [file 41419_2021_3641_MOESM2_ESM.tif]

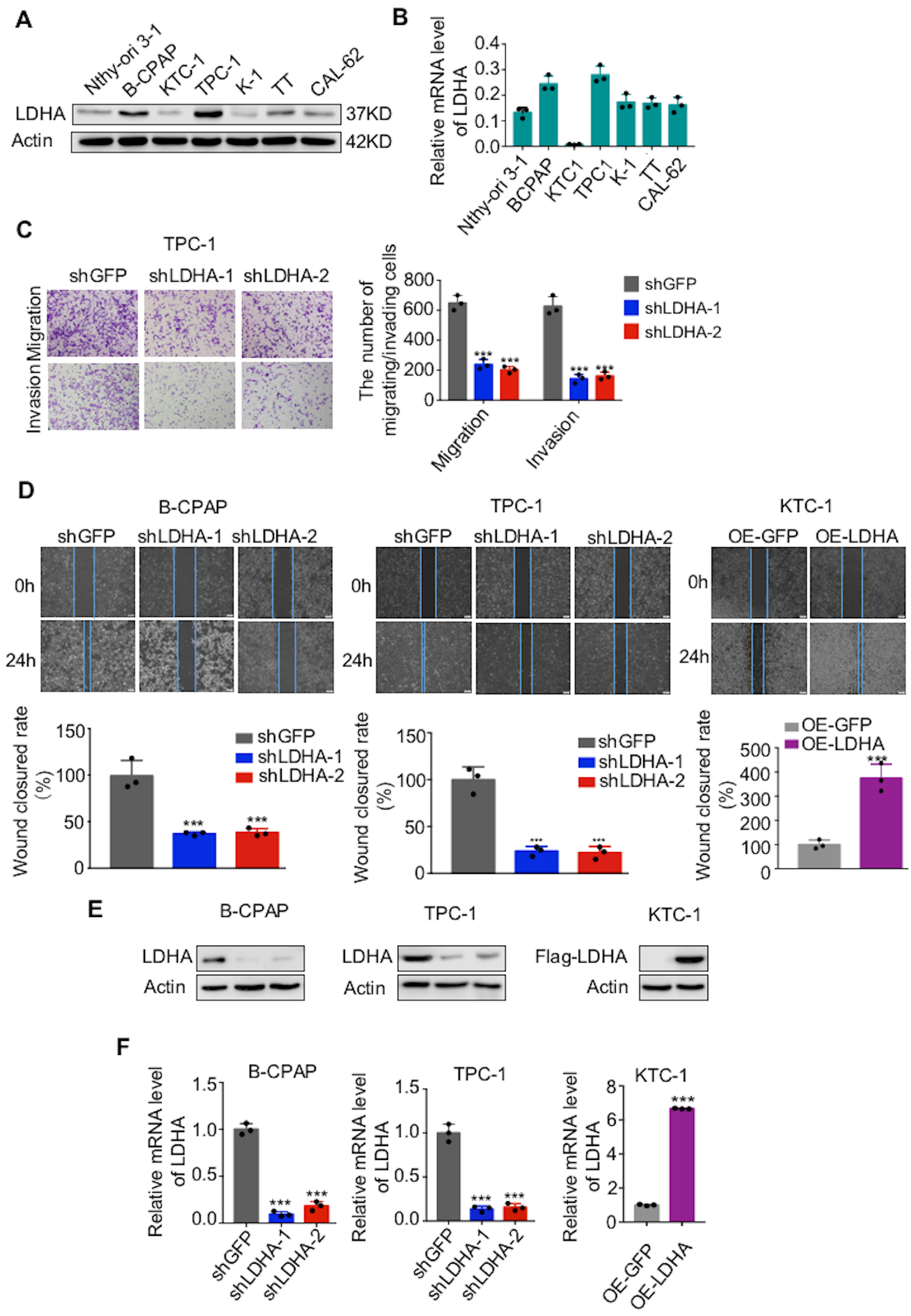

Supplement: Supplementary file 3 — Figure S3 [file 41419_2021_3641_MOESM3_ESM.tif]

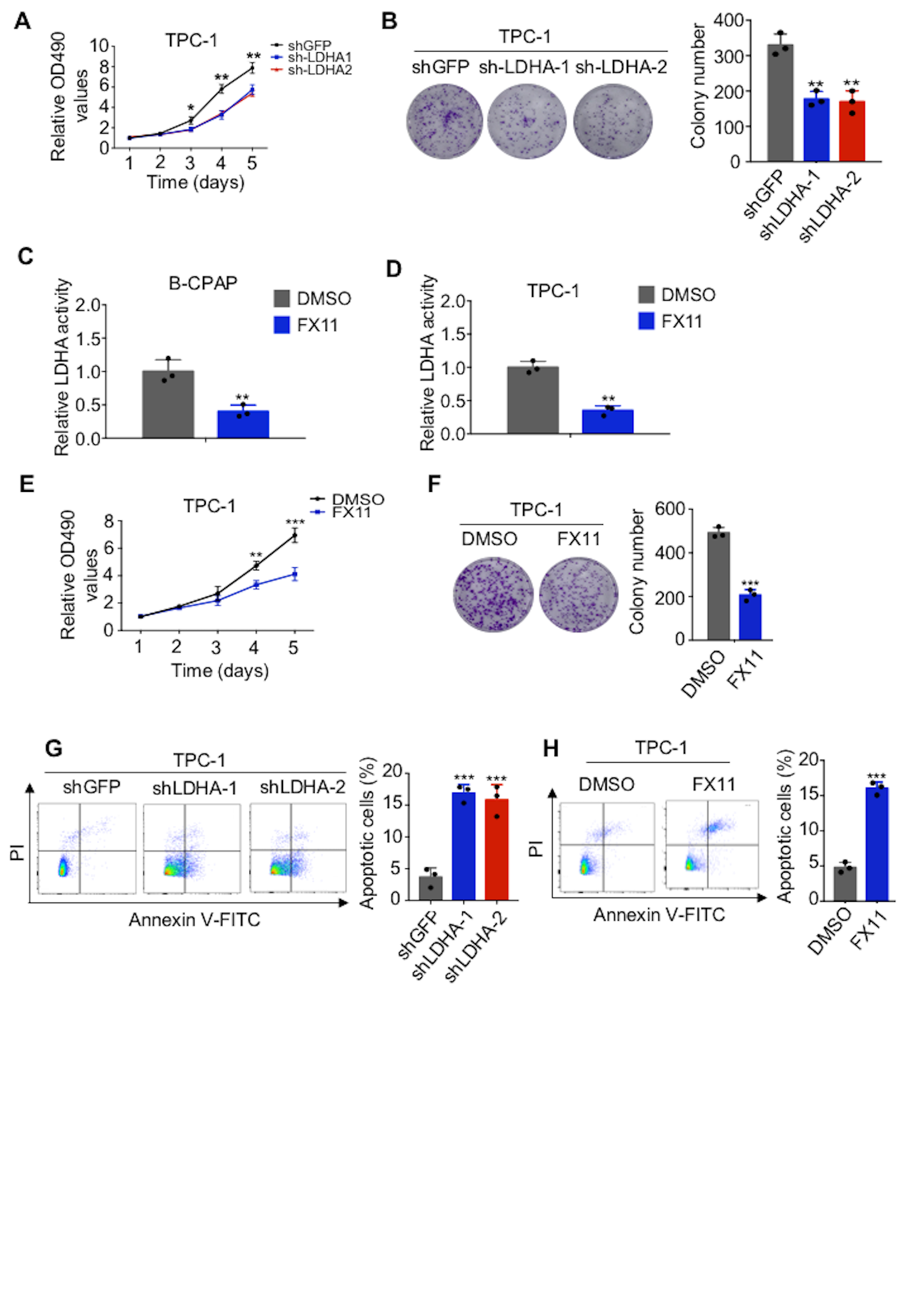

Supplement: Supplementary file 4 — Figure S4 [file 41419_2021_3641_MOESM4_ESM.tif]

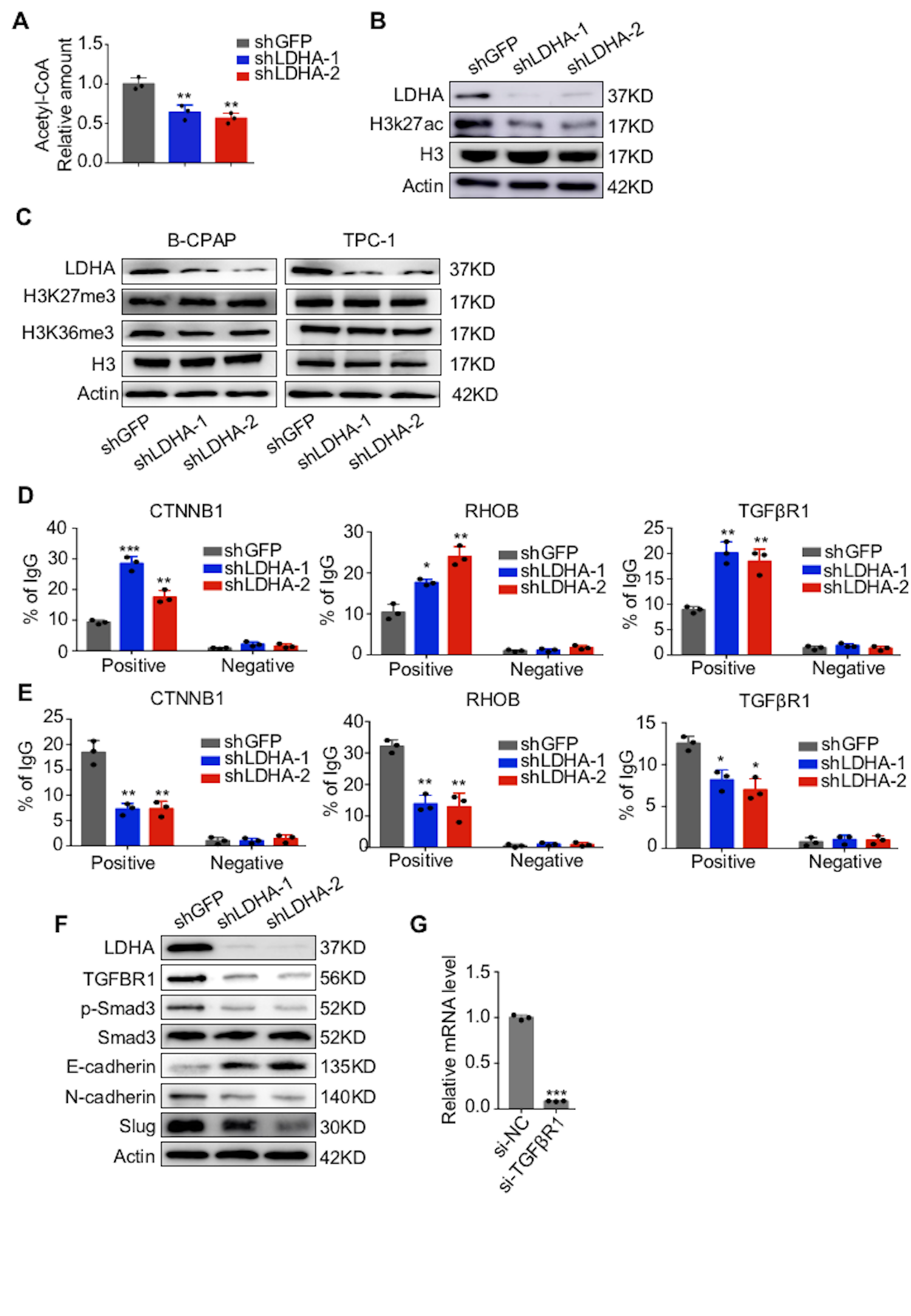

Supplement: Supplementary file 5 — Figure S5 [file 41419_2021_3641_MOESM5_ESM.tif]

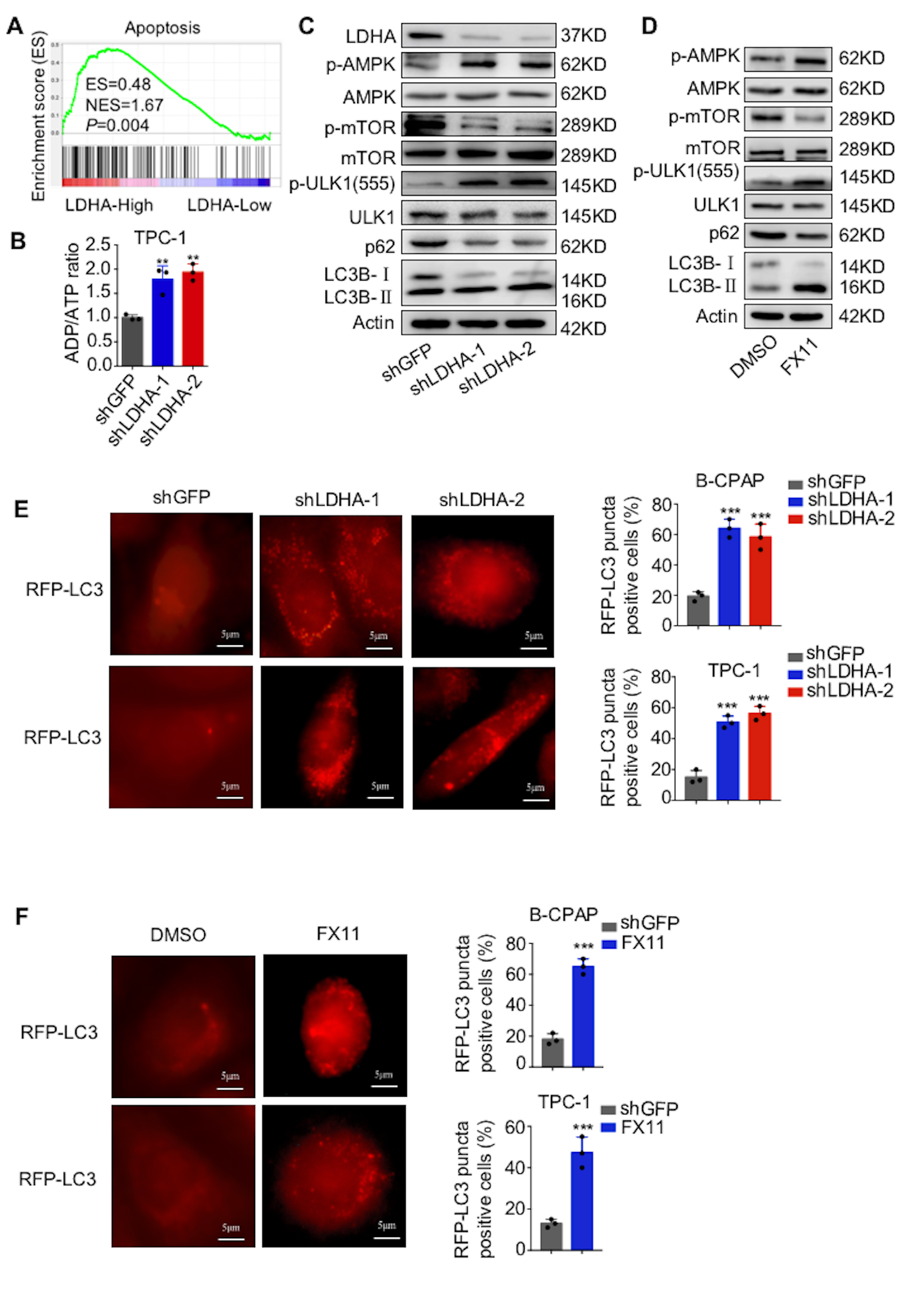

Supplement: Supplementary file 6 — Figure S6-1 [file 41419_2021_3641_MOESM6_ESM.tif]

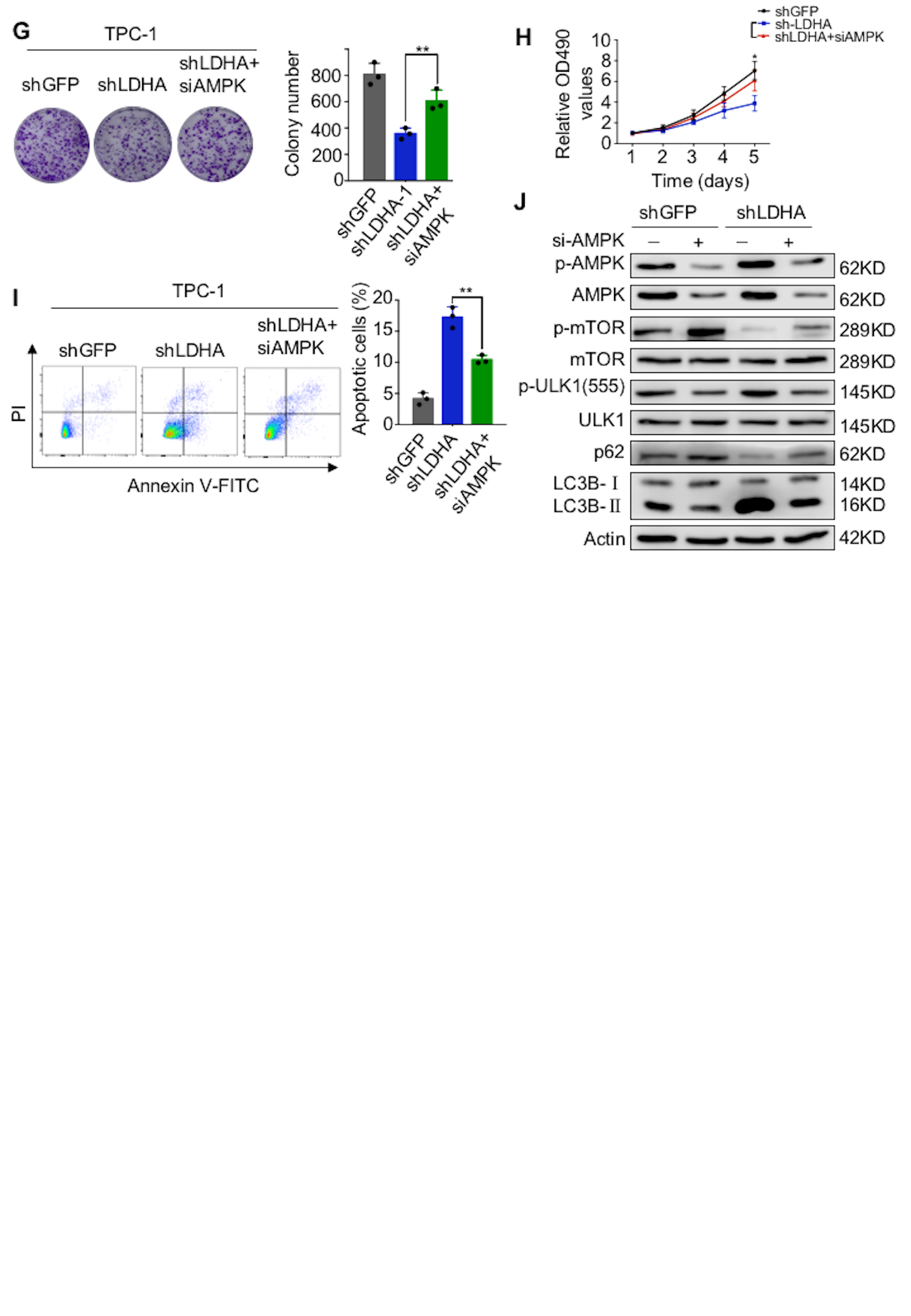

Supplement: Supplementary file 7 — Figure S6-2 [file 41419_2021_3641_MOESM7_ESM.tif]

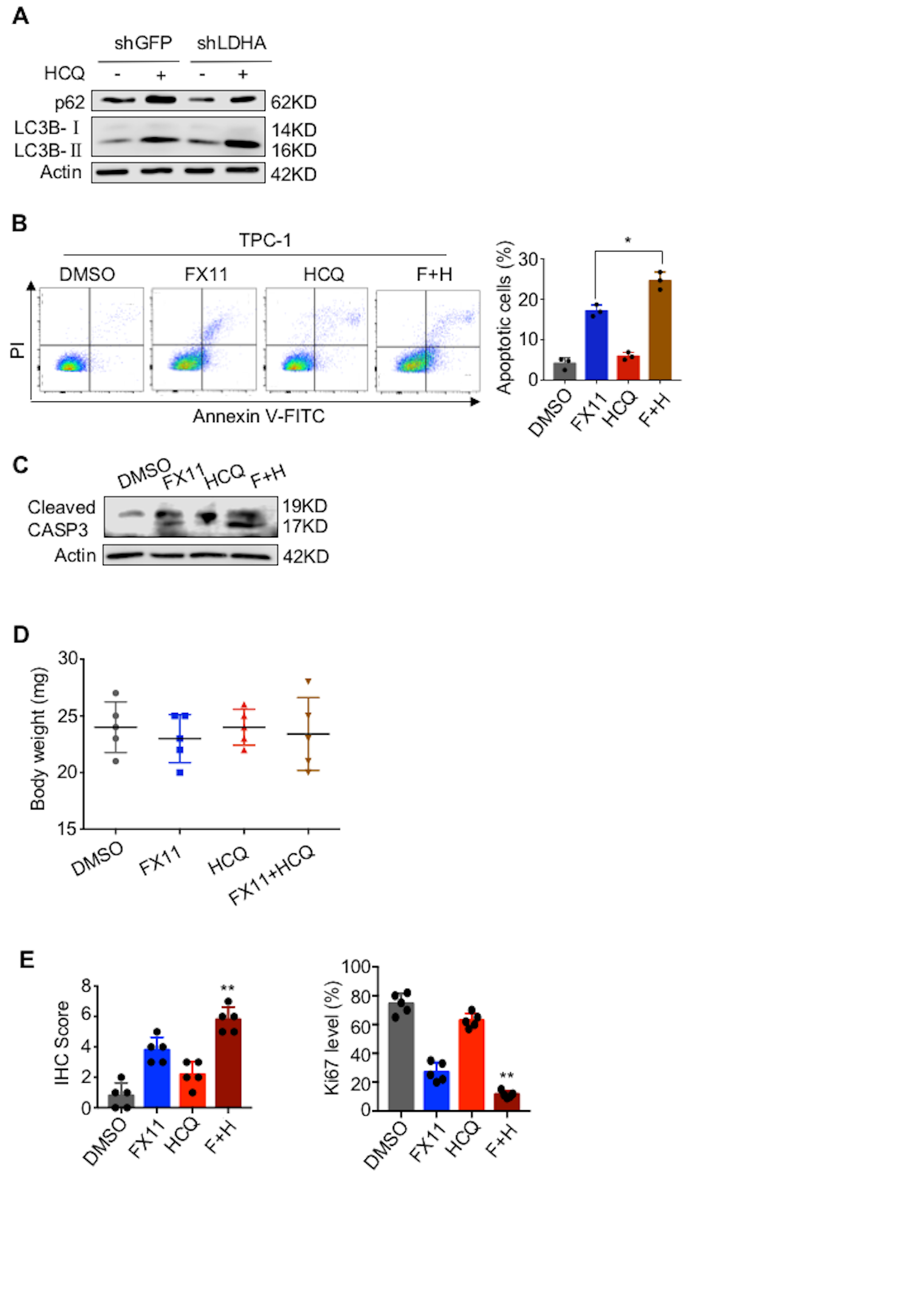

Supplement: Supplementary file 8 — Figure S7 [file 41419_2021_3641_MOESM8_ESM.tif]
