# Supplementary material for: LDHA induces EMT gene transcription and regulates autophagy to promote the metastasis and tumorigenesis of papillary thyroid carcinoma
Source: Cell Death Dis. 2021 Apr 1;12(4):347. doi: 10.1038/s41419-021-03641-8 (PMC8017009; doi:10.1038/s41419-021-03641-8)
Supplement: Supplementary file 9 — Supplementary Information [file 41419_2021_3641_MOESM9_ESM.docx]

**LDHA induces EMT gene transcription and regulates autophagy to promote the metastasis and tumorigenesis of papillary thyroid carcinoma**

Xiukun Hou, Xianle Shi, Wei Zhang, Dapeng Li, Linfei Hu, Jihong Yang, Jingzhu Zhao, Songfeng Wei, Xi Wei, Xianhui Ruan, Xiangqian Zheng, and Ming Gao

**Supplementary Figures**

**Fig S1 Bioinformatics analysis of enzymes involved in glucose metabolism and identifies LDHA as a candidate target for PTCs.** (A) The expression of genes involved in glucose metabolism in primary tumor tissues verse normal tissues. (B) Patients with extrathyroidal extension had higher LDHA levels than those without extrathyroidal extension. All **p*<0.05, ** *p*<0.01, *** *p*<0.001.

**Fig S2 The expression of LDHA in PTCs and Kaplan–Meier estimates of the probability of OS and RFS in PTC patients.** (A) Densitometric analysis showed a higher LDHA level in tumor tissues than in normal tissues when normalized to the actin expression level.

**Fig S3 LDHA promotes the migration and invasion of PTC cells *in vitro* and *in vivo*.** Protein (A) and mRNA (B) expression levels of LDHA in common thyroid carcinoma cell lines. (C) Representative images of migrating and invading TPC-1 cells with LDHA knockdown. (D) Cell migration was analyzed by wound-healing assay in B-CPAP and TPC-1 cells with LDHA knockdown and in KTC-1 cells expressing LDHA. (E-F) Knockdown and overexpression of LDHA in B-CPAP, TPC-1, and KTC-1 cells were detected by western blot and qRT-qPCR assays, respectively. Actin was used for the normalization in the qRT-PCR assay. All **p*<0.05, ** *p*<0.01, *** *p*<0.001.

**Fig S4 LDHA promotes the proliferation and inhibits the apoptosis of PTC cells *in vitro* and *in vivo*.** CCK-8 assay (A) and colony formation assay (B) showed that LDHA knockdown inhibited the proliferation of TPC-1 cells. (C-D) LDHA activity assay of B-CPAP and TPC-1 cells treated with DMSO or FX11 (10 μM for 24 h). The CCK-8 assay (E) and colony formation assay (F) showed that FX11 (10 μM for 24 h) attenuated the proliferation of TPC-1 cells. (G-H) Flow cytometry indicated that TPC-1 cells treated with LDHA knockdown or FX11 (10 μM for 24 h) induced apoptosis. The data are presented as the mean ± SD. All **p*<0.05, ** *p*<0.01, *** *p*<0.001.

**Fig S5 LDHA promotes the transcription of genes involved in the EMT process.** (A) Intracellular acetyl-CoA levels were measured in TPC-1 cells treated with LDHA knockdown. (B) H3K27ac levels were decreased after LDHA knockdown in TPC-1 cells. (C) Protein levels of H3K27me3 and H3K36me3 in B-CPAP and TPC-1 cells after LDHA knockdown. ChIP-qPCR assays were performed in B-CPAP cells using H3K27me3 (D) and H3K36me3 (E) antibodies to determine the expression levels of CTNNB1, RHOB, and TGFβR1. (F) Western blot assay showed the changes of TGFβR1, phosphorylated Smad3, E-cadherin, N-cadherin, and Slug in TPC-1 cells with LDHA knockdown. All *p<0.05, ** p<0.01, *** p<0.001. (G) Knockdown of TGFβR1 with siRNA in KTC-1 cells expressing LDHA was evidenced by qRT-PCR. All **p*<0.05, ** *p*<0.01, *** *p*<0.001.

**Fig S6 LDHA regulates tumorigenesis and autophagy through the AMPK signaling pathway.** (A) Gene expression data acquired from the TCGA database were subjected to GSEA v2.2.0, and the results showed that LDHA expression was correlated with the apoptosis-related pathway. (B) The ADP/ATP ratio was measured in TPC-1 cells treated with LDHA knockdown. (C-D) Western blotting was used to detect phosphorylated AMPK, phosphorylated mTOR, phosphorylated ULK1, p62, and LC3BII/I in TPC-1 cells treated with LDHA knockdown or exposed to 10 μM of FX11 for 24 h. (E and F) B-CPAP and TPC-1 cells transfected with RFP-LC3 were treated with LDHA knockdown or exposed to 10 μM of FX11 for 24 h. Fluorescence images were obtained by confocal microscopy. Colony formation assay (G) and CCK-8 assay (H) showed that the knockdown of AMPK partially attenuated the inhibited proliferation induced by LDHA knockdown in TPC-1 cells. (I) Flow cytometry indicated that the knockdown of AMPK partially reversed the apoptosis induced by LDHA knockdown in TPC-1 cells. (J) TPC-1 cells stably knocking down LDHA were infected with siRNAs targeting AMPK. Western blotting was used to detect phosphorylated AMPK, phosphorylated mTOR, phosphorylated ULK1, p62, and LC3BII/I expression levels. The data are presented as the mean ± SD. All **p*<0.05, ** *p*<0.01, *** *p*<0.001.

**Fig S7 HCQ enhanced the antitumor effect of FX11 *in vitro* and *in vivo*.** (A) TPC-1 cells with stable LDHA knockdown were treated with the autophagy inhibitor hydroxychloroquine (HCQ). Western blotting was used to detect p62 and LC3BII/I levels. (B) Apoptotic TPC-1 cells were detected by flow cytometry after exposure to 10 μM of FX11 for 24 h with/without HCQ (10 μM for 24 h). The data are presented as the mean ± SD. (C) TPC-1 cells were exposed to FX11 (10 μM for 24 h) with/without HCQ (10 μM for 24 h), and western blotting was used to detect cleaved caspase 3. (D) FX11 (3 mg/kg) with/without HCQ (60 mg/kg) was given to mice bearing papillary thyroid tumors for 21 days. Mice weights were measured on day 30. (E) IHC score of cleaved caspase 3 expression and Ki-67 staining of xenografted tumor specimens from the FX11 with/without HCQ groups are shown. All *p<0.05, ** p<0.01, *** p<0.001.

**Supplementary Table 1**

**Univariate analysis of clinicopathological features and LDHA expression**

|  |  | | |  | | | LDHA expression | | | |  | | |  | |
| --- | --- | --- | --- | --- | --- | --- | --- | --- | --- | --- | --- | --- | --- | --- | --- |
| Variables | Total | | | Low expression | | | High expression | | | | X^2^ | | | *P* | |
|  |  | | | ( n = 105) | | | ( n = 80) | | | |  | | |  | |
| Age |  |  | | |  | | |  |  | | | |  | |  |
| <55 | 69 | | | 41 | | | 28 | | | | 0.459 | | | 0.498 | |
| >=55 | 116 | | | 63 | | | 53 | | | |  | | |  | |
| Sex |  | | |  | |  |  | | |  | |  | |  | |
| Female | 132 | | | 76 | | | 56 | | | | 0.346 | | | 0.556 | |
| Male | 53 | | | 28 | | | 25 | | | |  | | |  | |
| Multifocality | | |  | | | |  | | |  | |  | |  | |
| Present | 58 | | | 34 | | | 24 | | | | 0.198 | | | 0.656 | |
| Absent | 127 | | | 70 | | | 57 | | | |  | | |  | |
| T stage | | |  | | |  |  | | |  | |  | |  | |
| T1/2 | 167 | | | 99 | | | 68 | | | | 6.552 | | | 0.010 | |
| T3/4 | 18 | | | 5 | | | 13 | | | |  | | |  | |
| N stage | | |  | | | | | | | |  | | |  | |
| N0 | 89 | | | 59 | | | 30 | | | | 7.074 | | | 0.008 | |
| N1a/b | 96 | | | 45 | | | 51 | | | |  | | |  | |
| TNM stage | | |  | | | |  | | |  | |  | |  | |
| I+II | 174 | | | 103 | | | 71 | | | | 10.552 | | | 0.001 | |
| III+IV | 11 | | | 1 | | | 10 | | | |  | | |  | |

**Supplementary Table 2**

**Multivariate COX regression analysis of RFS and OS in relation to clinicopathological features**

| Variables | Recurrence-free survival |  | Overall survival |  |
| --- | --- | --- | --- | --- |
|  | Hazard ratio (95% CI) | *P* | Hazard ratio (95% CI) | *P* |
| Age>=55  Female | 0.563 (0.235 – 1.349)  1.486 (0.558 – 3.958) | 0.198  0.428 | 0.486 (0.223 – 1.062)  1.753 (0.617 – 4.977) | 0.292  0.292 |
| III+IV | 4.950 (1.299 – 18.864) | 0.019 | 5.414 (1.389 – 21.096) | 0.015 |
| Multifocality | 1.075 (0.429 – 2.692) | 0.878 | 0.593 (0.211 – 1.664) | 0.321 |
| LDHA expression | 4.848 (1.890 – 12.44) | 0.010 | 5.545 (2.026 – 15.175) | 0.001 |

**Supplementary Table 3**

**The qRT-PCR primer sequences were as follows:**

| Gene | Primer | Sequence |
| --- | --- | --- |
| LDHA | Forward | CGTGTTATTGGAAGCGGTTG |
|  | Reverse | TTCATTCCACTCCATACAGGC |
| ZEB1 | Forward | CCTGTGAATGGGCGACCAAGAAC |
|  | Reverse | GGACTGCCTGGTGATGCTGAAAG |
| RHOB | Forward | CGAGCATGTCCGCACAGAGC |
|  | Reverse | GCACGCCTTCCTTGGTCTTGG |
| CDH2 | Forward | GGACAGTTCCTGAGGGATCAAAGC |
|  | Reverse | CTTGGAGCCTGAGACACGATTCTG |
| FN1 | Forward | GGCGACAGGACGGACATCTTTG |
|  | Reverse | GGCACAAGGCACCATTGGAATTTC |
| TPM1 | Forward | CGACAAGAAGGCGGCGGAAG |
|  | Reverse | CCAGTTCATCTTCGGTGCCCTTG |
| TGFβR1 | Forward | GCAGAGCTGTGAAGCCTTGAGAG |
|  | Reverse | ATGCCTTCCTGTTGACTGAGTTGC |
| TGFβR2 | Forward | TAACCTGCTGCCTGTGTGACTTTG |
|  | Reverse | TCCCACCTGCCCACTGTTAGC |
| CTNNB1 | Forward | CCTCCCAAGTCCTTTATGAATGG |
|  | Reverse | CCGTCAATATCAGCTACTTGCTCTT |
| NDUFA4L2 | Forward | GAGCCCTGGAACCGCCTGAG |
|  | Reverse | AGTCTGGCCGGTCCTTCTTCAG |
| NDUFA3 | Forward | GTGCTGGTCGTGTCCTTCGTC |
|  | Reverse | ACGGGCACTGGGTAGTTGTAGG |
| NDUFB1 | Forward | GGGACCACTGGGTTCATGTTCTTG |
|  | Reverse | CGGAAGGCAGTTAGCCGTTCATC |
| β-actin | Forward | GATCATTGCTCCTCCTGAGC |
|  | Reverse | AC TCCTGCTTGCTGATCCAC |
| CTNNB1-Positive | Forward | ACTACTTTCCACCGCCCCCT |
|  | Reverse | CTGCTGCCACAGACCGAGAG |
| CTNNB1-Negative | Forward | ACAGCAATCAGCTGGCCTGG |
|  | Reverse | CCACTCCCACCCTACCAACC |
| RHOB-Positive | Forward | CCAATGTGCCCATCATCCTG |
|  | Reverse | TCGAGGTAGTCGTAGGCTTG |
| RHOB-Negative | Forward | GTCCCTTTGAGAGCATGTGG |
|  | Reverse | AGGAATGTGACAGGAACAACC |
| TGFβR1-Positive | Forward | TTCTTCTTTCGTGCGTCTGG |
|  | Reverse | AAACCCGCAGAATGAGCAAA |
| TGFβR1-Negative | Forward | TGGCCAGTTGTATCGCCTAT |
|  | Reverse | TGGCACTGTACACCAACTCA |
